# Food policy, practice and provision in UK early childhood education and care: a scoping review

**DOI:** 10.1017/S1368980025101298

**Published:** 2025-10-13

**Authors:** Amy Turner, Alice Porter, Marie Murphy, James Parsonage, Joseph Coombes, Ruth Kipping

**Affiliations:** 1 Centre for Public Health, Population Health Sciences, Bristol Medical School, University of Bristol, Bristol, UK; 2 National Institute for Health and Care Research (NIHR) School for Public Health Research (SPHR), London, UK; 3 NIHR Bristol Biomedical Research Centre, University Hospitals Bristol and Weston NHS Foundation Trust and University of Bristol, Bristol, UK; 4 Institute of Applied Health Research, University of Birmingham, Birmingham, UK

**Keywords:** Children, Food Practices, Food Policy, Nutrition, Review

## Abstract

**Objective:**

To evaluate research exploring food policy, practice and provision in early childhood education and care (ECEC) settings, using the socio-ecological model (SEM).

**Design:**

A scoping review was conducted in accordance with the Joanna Briggs Institute (JBI) Methodology for scoping reviews and the Preferred Reporting Items for Systematic Reviews and Meta-analysis for Scoping Reviews (PRISMA-ScR). Five databases were systematically searched. Eligible studies were retrieved after full-text screening. Data were extracted and synthesised based on food policy, practice and provision concepts and grouped according to SEM level. Results were presented using a narrative summary.

**Results:**

Twenty-four studies were included, the majority had qualitative (*n* 13, 54 %) or cross-sectional study designs (*n* 11, 46 %) and presented findings at the organisational SEM level. Nursery settings were most represented (*n* 16, 67 %), followed by childminders (*n* 5, 21 %), then preschools (*n* 3, 13 %). Studies were conducted in England (*n* 20, 83 %) and Scotland (*n* 2, 8 %); however, no studies were undertaken in Wales or Northern Ireland. Studies reported poor adherence to food policies in ECEC. Recommended practices were frequently adhered to; however, food provided did not consistently meet nutritional recommendations. Common barriers to implementing healthy food practices and provision were cost, staff shortages, lack of training and awareness of available guidance.

**Conclusions:**

This review identified a need for research that targets a range of SEM levels and is conducted in Scotland, Wales and Northern Ireland. Our findings support the need for increased governmental support for ECEC, through food standards, free meal provision for ECEC and more accessible nutrition training.

Reducing child overweight and obesity is an international priority^(1)^. In England, 22 % of 4- to 5-year-olds were overweight or living with obesity in 2023/24^(2)^, comparably high levels are reported across Scotland, Northern Ireland and Wales^(3–5)^. Notably, children living in the most deprived areas are more likely to be living with obesity in England and Wales^(2,5)^. Therefore, promotion of healthy weight in early years children (0–5 years) is a public health priority in all four UK countries (England, Scotland, Northern Ireland and Wales)^(6–8)^.

Dietary intake during early childhood is a modifiable risk factor for obesity^(9)^. Unhealthy dietary patterns, high in energy-dense foods and low in fibre, are associated with increased adiposity and obesity risk in childhood and adolescence^(10–12)^. Studies have also shown that healthy dietary patterns in early childhood have long-term protective effects against stroke and cancer risk and are associated with better cognitive outcomes^(13–16)^. Despite this, recent evidence has indicated that dietary intake in young children in the UK often does not meet nutritional recommendations for fibre, Zn and vitamin C^(17,18)^.

In Scotland, 79 % of households with early years children used some form of childcare in 2019^(19)^, and on average children aged 0–4 years spent 22·5 h a week in formal childcare in England in 2023^(20)^. Formal childcare refers to early childhood education and care (ECEC) settings such as nurseries, preschools and childminders that provide daytime care for children before they reach school age (4–5 years). Food consumed during ECEC comprises 11 % of total eating occasions among early years children in the UK^(21)^. ECEC could therefore be a pivotal setting to influence dietary behaviours and nutritional intake in early years children to help mitigate impacts of obesity and diet-related outcomes in children.

Scotland, Wales and England have government-funded schemes where parents can claim up to 30 h of free childcare to make childcare more economically feasible for parents^(22)^. Northern Ireland also supports parents through The Northern Ireland Childcare Subsidy Scheme (NICSS) and the Tax-Free Childcare (TFC) scheme^(23,24)^. Recent increases (2024) to the funded childcare hours in England and the NICSS scheme are estimated to increase demand for childcare by 15 %^(25,26)^. The impact increased demand for childcare could have on feeding practices and food provision in ECEC is unknown as funding for meals is not included within the free childcare hours scheme in England^(27)^. However, meals are free to children attending ECEC in Scotland^(28)^. The upcoming changes to childcare provision in England may also have a disproportionate impact in the most deprived areas, where ECEC already face chronic underfunding, and children have poorer dietary outcomes^(29–31)^. Thus, the aim of this scoping review was to draw together all current UK-based evidence on food policy, practice and provision in ECEC to identify gaps that will inform future policy development and research. Our review will address the following research questions: (i) how is food policy and guidance used by ECEC settings?; (ii) what food practices are used in ECEC settings in the UK?; and (iii) does food provided and consumed within ECEC settings meet nutritional recommendations for children of 0–5 years old?

The secondary aim of this review was to map findings from the included studies to the socio-ecological model (SEM) to identify how food policies, feeding practices and food provision in UK ECEC have been explored in research. The SEM is based on the ecological systems theory^(32)^ which hypothesised that individual, interpersonal, organisational, environmental and governmental contexts should be considered to fully understand individual outcomes^(33,34)^.

## Methods

This scoping review was conducted in accordance with the JBI methodology for scoping reviews^(35)^ and was reported using the PRISMA extension for scoping reviews checklist (available in Appendix 1)^(36,37)^. A protocol for this scoping review was pre-registered on Open Science Framework (DOI 10.17605/OSF.IO/Q2RPH) and remained unaltered throughout the review process.

### Search strategy

A search strategy was developed using terms for key concepts of the review: ‘early years care’, ‘nutrition’, ‘feeding practices’, ‘food events’ and ‘nutrition policy’. The terms were combined using ‘OR’ and then grouped using ‘AND’ to link the concepts. Limits were applied to restrict results to the English language and research published after 1990, as childhood obesity prevalence in the UK had a marked increase after 1994^(38)^; therefore, research published before this time may have less relevance to current public health policy. After a pilot search was carried out, the strategy was reviewed by a subject librarian to ensure the search retrieved all relevant studies. The search was performed in MEDLINE, EMBASE, PsycINFO, Scopus and CINAHL databases, in May 2024. The search strategies used are available in Supplementary material 2.

### Eligibility criteria and study selection

The inclusion criterion for this scoping review was based on the Population, Concept, Context framework^(35)^. Studies were eligible for inclusion if (i) the population included early years children aged 0–5 years, parents, early years practitioners and/or staff; (ii) they explored the concept of food policy, feeding practices and/or food provision in ECEC (Table 1); and (iii) and if they were conducted in the context of UK ECEC.

**Table 1:** Concepts of food policy, practice and provision eligible for inclusion in the review grouped by SEM level

SEM level	Individual	Interpersonal	Organisational	Environmental	Governmental
Policy		-Lunchbox policies for parents	-ECEC healthy eating policies -Voluntary food and drink guidance	-Local council food policies -Deprivation and socio-economic factors affecting policy use -Local healthy eating schemes for early years	-Governmental public policies -Government schemes -Ofsted monitoring
Practice		-Interaction with parents -Parental demographic factors -Peer influence	-Eating environment -Use of appropriate utensils -Staff nutrition education -Awareness and use of voluntary food guidelines -Serving styles in ECEC -ECEC eating with child and role modelling	-Cultural influences -Deprivation and socio-economic factors affecting feeding practices in ECEC	-Government funding impact on practices
Provision	-Child preferences -Fussy eating -Child self-serving	-Lunchboxes/food from home served in ECEC	-Nutrition of food provided in ECEC -Food preparation method: internal chef, catering company, school canteen	-Food insecurity -Economic barriers -Spatial factors affecting source of food -Deprivation and socio-economic factors affecting provision	-Government-funded initiatives providing food to ECEC

SEM, socio-ecological model; ECEC, early childhood education and care.

Articles were excluded if they were:

1. Conducted outside the four UK countries (England, Scotland, Wales and Northern Ireland).
2. Review articles, protocols, commentaries, opinion pieces and grey literature.
3. Focused on food in afterschool, breakfast or holiday clubs.
4. Focused on children with chronic or medical conditions and/or special feeding requirements.

**Figure 1.**
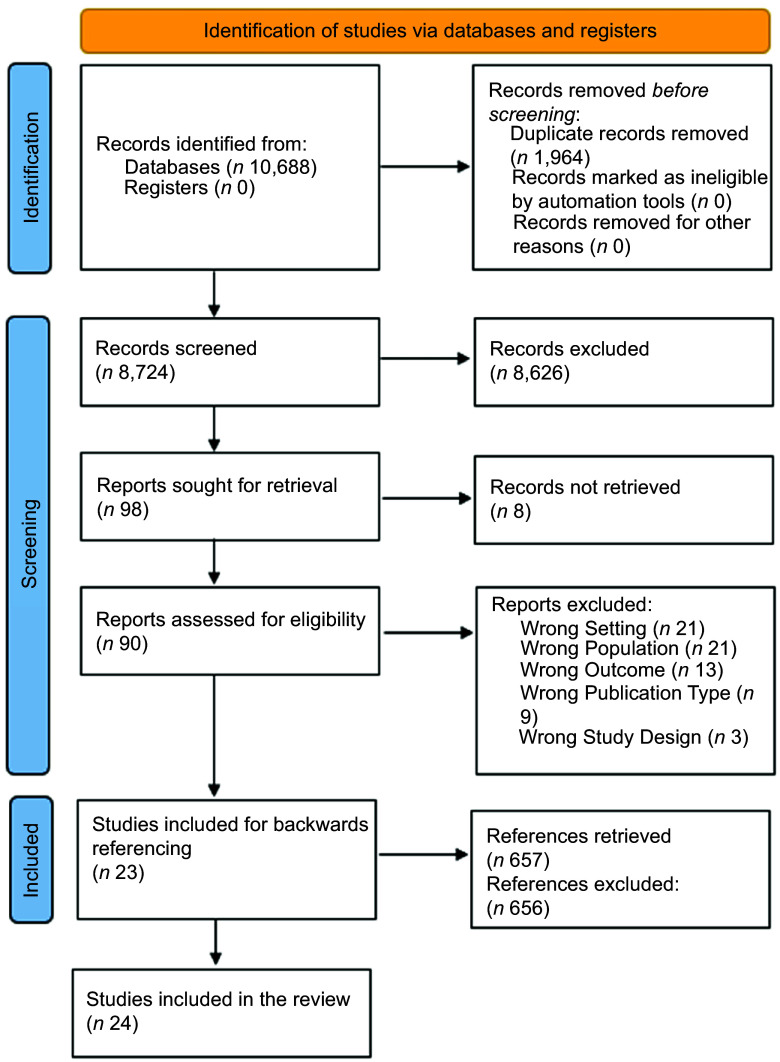
PRISMA 2020 flow diagram representing identification of articles from database searching and screening process.

All titles and abstracts retrieved from the database searches were exported into EndNote to remove duplicate records and then uploaded into Rayyan screening software for screening^(39)^. Three reviewers (AT, JP and JC) independently screened all titles and abstracts for inclusion in full-text screening. All full texts were then screened independently by the first reviewer (AT), and a random 50 % were independently screened by a second reviewer (JP), as previously deemed adequate^(40)^. Discrepancies were discussed with the research team. Reference lists of the included studies were identified using CitationChaser and screened by the first reviewer (AT). Reasons for exclusion of articles at the full-text screening stage are reported in Figure 1.

### Data charting

A data extraction form was created in Excel and was designed to capture methodological characteristics of the studies and outcomes related to the target population, concept and context. The data extraction form was piloted with five studies by two reviewers to assess suitability. After appropriate adjustments were made, the first reviewer (AT) independently extracted all relevant data from the studies, and a second reviewer (JP) extracted a random selection of 50 % of the studies to ensure consistency. Discrepancies were discussed and resolved by the two reviewers.

### Data synthesis, analysis and presentation

Characteristics of the studies such as type of participant, geographical information and type of setting were extracted and summarised in Table 2. Key findings identified from the studies were summarised and categorised based on relevance to the key concepts of the review: food policy, practice or provision. Additionally, results from studies were grouped according to SEM levels (individual, interpersonal, organisational, environmental and governmental). A narrative summary approach was used to discuss findings from the studies, and this did not include assessment of methodological quality of the included studies, as appropriate with the scoping review design^(35)^.

**Table 2:** Summary of study characteristics

	*n*	%	Number of studies	%
Type of participant				
Child	1906	34·9	5	20·8
ECEC staff	542	9·9	12	50·0
ECEC manager	1543	28·2	7	29·2
ECEC owner	6	0·1	2	8·3
Cook	14	0·2	3	12·5
Childminder	82	1·5	3	12·5
Parent	1353	24·7	9	37·5
Other^*^	21	0·4	2	8·3
Total	5467			
Country				
England			20	83·3
Wales			0	
Scotland			2	8·3
Northern Ireland			0	
Multiple UK nations			2	8·3
Region England				
Yorkshire			5	25
North West			3	15
South East			2	10
South West			2	10
London			1	5
East Midlands			1	5
North East			1	5
East			0	
West Midlands			0	
Multiple regions			5	25
Type of early years setting				
Nursery			16	66·7
Preschool			3	12·5
Childminders			5	20·8
Setting characteristics				
Private			7	
Local authority-funded			7	
School-based			4	
Not available			12	

ECEC, early childhood education and care.

* Other types of participants included non-government organisations (NGOs), member organizations, local authority stakeholders, health visitors and researchers.

**Table 3:** Summary of recommendations and actions for policy and research identified by study authors

	Future research designs	Feeding practices related	Food provision related
Recommendations for research	-Need for larger ethnographic studies that include higher number of nurseries and childminders^(54)^. -Future study samples should be purposefully selected to reflect the ratio of childcare providers to provide better applicability to local context^(49)^.	-Further research to understand factors influencing nurseries’ use of guidelines and views on whether guidelines should be voluntary or statutory^(60)^. -Future research should capture actual practice as well as reported^(49)^.	-Need to identify deficiencies in nutritional quality of food being consumed in EYS^(52)^. -Offering vegetables to children at breakfast was feasible to implement, and a definitive randomised controlled trial should be undertaken^(42)^.
	Food policy-related	Feeding practices-related	Food provision-related
Recommendations for policy	-Need for authoritative set of dietary guidelines for early years that are accessible to all EYS^(53,56)^. -Should develop educational and promotional public health campaigns for portion size guidance^(57)^. -Recommend improved consultation with EYS sector and sustainable investment in EYS^(55)^. -Recommend development of a standard healthy eating policy for all EYS^(48)^. -Need to understand how policies can best be communicated to parents and incorporate parental views into policy development^(49,51)^.	-Further support and training for developing menu ideas, hands-on food training and use of portion size guidance^(48,50,57)^. -Active engagement regarding breast-feeding policies in EYS^(104)^. -Settings should have more opportunities to share knowledge and practice with range of health professionals^(44)^. -Evidence-based guidance and practical tools to facilitate effective practice must be provided to EYS^(49)^. -Need for universal national training programme for healthy eating practices in EYS^(48)^.	-Recommendation for increased support for schools and caterers providing food for multiple age groups^(63)^.

## Results

Out of 10 688 articles retrieved, 1964 duplicates were removed, and 90 full-text articles were assessed for eligibility after title and abstract screening. Following full-text screening, twenty-three articles were included (Figure 1). One additional article was identified from the reference lists of included articles.

### Characteristics of studies

The majority (*n* 20, 83 %) of included studies were conducted in England, and the remaining studies were conducted in Scotland (*n* 2, 8 %) or were UK wide (*n* 2, 8 %); there were no studies conducted in Northern Ireland or Wales (Table 2).

Thirteen studies (54 %) utilised qualitative methods such as semi-structured interviews. Studies also utilised cross-sectional study designs (*n* 11, 46 %), and three studies (13 %) used experimental designs, two of which were mixed-method evaluations of interventions^(41,42)^, and one was a cluster randomised controlled trial^(43)^.

The majority of studies included ECEC staff (*n* 12, 50 %), followed by parents (*n* 9, 38 %) and then ECEC managers (*n* 7, 29 %). Despite the greatest proportion of participants being early years children, only five studies (21 %) included children as participants^(42–46)^. Most commonly, studies were based in nurseries (*n* 16, 67 %) (Table 2).

**Figure 2.**
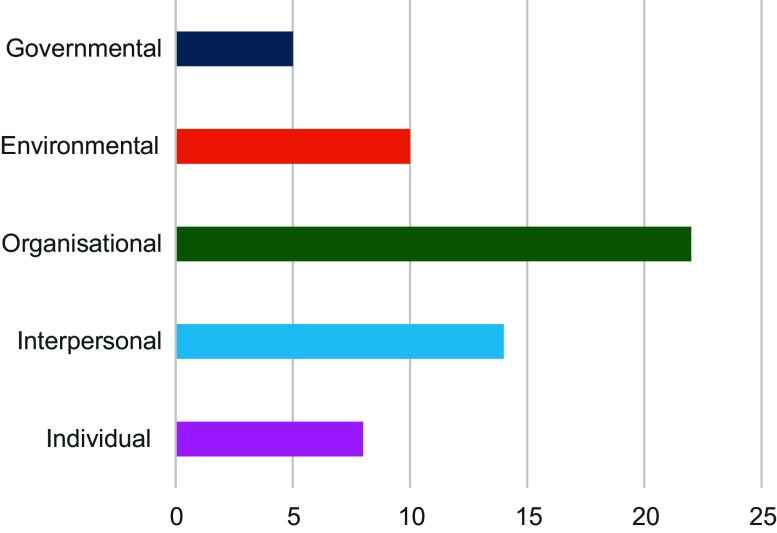
Number of studies that investigated each SEM level. SEM, socio-ecological model.

Figure 2 displays the proportion of studies that addressed each SEM level. The most frequent SEM level addressed was the organisational level (*n* 22, 92 %); these studies often investigated staff feeding practices within ECEC, such as knowledge and use of food guidance, serving style and staff role modelling. The second most frequent SEM level addressed was the interpersonal level (*n* 14, 58 %), explored through parent–provider relationships, such as parental input into menu planning or parent engagement with food provided in ECEC. Most frequently, studies addressed two SEM levels, and no studies addressed all five SEM levels.

The narrative summary of results was explored further by ‘Policy’, ‘Practice’ and ‘Provision’ concepts. Furthermore, this review identified and summarised study author recommendations for food provision-related policy development and future research for each concept (Table 3).

### Policy

Food policy was the least investigated area compared to practice and provision. Fourteen studies (58 %) presented findings related to food policy.

#### Early childhood education and care food policies

Nine studies (38 %) investigated the implementation and adherence of ECEC food policies. The proportion of ECEC that had food policies differed between studies; two studies reported that 68–77 % of ECEC had a food policy^(47,48)^, whereas other studies reported much lower proportions of 13–17 %^(49,50)^. ECEC food policies reported in the studies included policies on staff roles during mealtimes or on the provision of ‘treats’^(49,51)^. However, ECEC food policies tended to have varying formats and content between settings^(49,52)^. Alderton and Campbell-Barr (2005) reported that settings which did not have a food policy tended to exhibit less positive food practice and provision outcomes, such as involving children in serving food and catering for special dietary needs^(47)^. Additionally, there were often varying degrees of policy enforcement from both providers and parents^(53)^. Policies surrounding ‘treats’ and celebrations were reported as difficult to enforce, and parental demand was a key influence upon adherence to policies^(51,52)^. The process evaluation of a dietary intervention in ECEC identified that the management structure and financial barriers can prevent ECEC from developing food policies^(41)^.

#### Availability of support and guidance to develop food policies

Several studies reported that there was less support and guidance available to private nurseries than local authority-funded nurseries^(49,50,54)^. According to findings from a qualitative study, staff from private nurseries felt they were more isolated from healthy eating information and policy guidance^(54)^. It was also reported that private settings relied on the Office for Standards in Education (Ofsted – government regulation body for educational settings in UK) requirements to guide feeding practices and food provision^(50)^ and did not receive the same support available to some local authority-funded nurseries^(49)^.

#### Impact of government-level food policy and schemes

Limited findings addressed the use or impact of local and national governmental policies or schemes. Studies that evaluated government-level policy were mostly qualitative and generally reported negative, critical, views of their impact in ECEC. Moore et al. (2005) reported that the introduction of Government Ofsted monitoring of childminders in England was viewed as damaging to relationships between childminders and the local authority and contributed to an increased reliance on food provided by parents, rather than by childminders^(52)^. Similarly, another qualitative study reported that ‘free childcare hours’ funded by government in England had put additional financial strain on ECEC, resulting in some ECEC relying on food provided by parents, or raising prices for parents not using the subsidy^(55)^. It was also reported that there was a low uptake of free milk provided as part of ‘The Nursery Milk Scheme’ to nurseries in England and Wales due to a preference from children for drinks brought from home^(44)^.

#### Recommendations for future policy development

Authors of the studies identified a need for authoritative, accessible guidance for food provision in ECEC and the implementation of a standardised food policy for ECEC in the UK^(48,53,56)^. Additionally, authors recommended promotional campaigns that target portion size guidance in ECEC^(57)^ and an increase in support and investment in the ECEC sector by UK governments^(55)^.

### Practice

The majority of findings from the studies related to feeding practices in ECEC (*n* 20, 83 %).

#### Feeding practices employed in early childhood education and care

Studies frequently reported the use of positive feeding practices in ECEC to support children throughout mealtimes, including giving gentle encouragement to try foods served and prompting children to eat their vegetables at mealtimes^(50,53,58)^. It was reported that one nursery enabled staff to eat for free with the children to promote role modelling^(41)^. Three studies reported that children were often encouraged to self-serve their food or involved in food preparation^(47,50,57)^. Child-sized tableware was used to guide appropriate portion sizes in ECEC, as one qualitative study reported that staff felt generally less aware of portion size guidance than parents^(57)^.

There was also evidence of feeding practices in ECEC which are not recommended by early years guidance. For example, 21 % of providers who responded to a questionnaire said that they used treats to incentivise children to eat their meal and encourage good behaviour^(58)^. Neelon et al. (2015) found that nurseries located in areas with the highest Index of Multiple Deprivation (IMD) score (i.e. most deprived) were more likely to report recommended practices such as allowing children to select their own food or staff accompanying children at mealtimes. However, there was generally an expectation children should finish all their food, which is not recommended practice^(59)^. Findings from the studies also indicated that ECEC rarely sought nutrition advice or approval from dietitians to support menu development^(48,50)^.

#### Barriers to healthy feeding practices

A common theme identified in seven studies was the lack of nutrition training and education of ECEC staff and cooks^(47,49,50,52–55)^. There was evidence that providers had some general healthy eating knowledge but often relied on ‘common sense’ or diet knowledge gained from personal experiences^(53)^. A process evaluation of an ECEC intervention found that there were small increases in ECEC staff healthy eating knowledge and motivation post-intervention, indicating that lack of healthy feeding training and education is a modifiable barrier^(41)^. Other barriers to healthy feeding practices identified in the studies included limited time, lack of kitchen facilities, lack of staff supporting mealtimes, and budgetary constraints^(41,47)^. Insufficient funding and budgetary concerns were prominent barriers to accessing training and support towards healthy feeding practices for ECEC^(55)^, and this was most evident in settings in more deprived areas^(41)^.

#### Use of early years food and drink guidance

Warren et al. (2024) reported that providers were more likely to be aware of healthy eating guidance than to use it in practice^(60)^. The most frequent sources of healthy eating information cited in the included studies were childminding magazines, ‘Change4Life’ materials, national reports and advice from parents^(49,53,59)^. Two studies found that the ‘Eat Better, Start Better’ voluntary food and drink guidelines for ECEC in England were a key source of information used by providers^(59,60)^. Notably, it was reported that providers frequently referenced feeding guidance aimed for school-aged children rather than early years children^(53)^. Barriers to the use and implementation of guidance in ECEC included the length and complexity of reports, and guidance not being culturally inclusive^(55)^. A quantitative study identified that settings with larger numbers of children were more likely to use guidelines^(60)^.

#### Recommendations for feeding practice-related policy development and future research

Three studies identified that further support and training for menu development, healthy food preparation and use of portion size guidance would be beneficial for ECEC^(48,50,57)^. It was also suggested that providers should actively engage with parents about feeding practices and seek opportunities to learn from a range of health professionals^(44)^.

Recommendations for future research included understanding the factors influencing the use of healthy feeding guidance and improving methods to more accurately capture feeding practices in a range of ECEC^(49,60)^.

### Provision

Fifteen studies (63 %) reported outcomes related to food provision in ECEC.

#### Food provided in early childhood education and care

Fruit and vegetables were frequently provided in ECEC^(48,59,61)^; however, ECEC did not often meet the oily fish recommendations (at least two portions per week)^(45,48,59,62)^. The provision of plant-based protein sources was also generally insufficient in ECEC, although nurseries in the most deprived areas were more likely to provide foods containing wholegrains, legumes, pulses and lentils^(59,63)^. Milk provision varied between ECEC, some settings provided semi-skimmed milk and some provided whole milk^(44)^. Studies reported that ECEC were likely to rely on processed foods or dried fruits, high in sugar, for snack foods^(50,53)^.

The provision of food brought from home in ECEC, referred to as ‘packed lunches’, was dependent on the setting type. Moore et al. (2005) reported that 25 % of childminders and 8 % of private nurseries relied on parents bringing food from home, whereas parents never provided food in the local authority-funded settings included in the study^(52)^.

#### Nutritional quality of food in early childhood education and care

A secondary data analysis using UK National Diet and Nutrition Survey (NDNS) data found that meals provided in nurseries and preschools were lower in added sugars and were less energy-dense than food provided by parents, wider family or other childcare^(46)^. There were conflicting findings reported regarding energy and carbohydrate content of food provided in ECEC^(48,63)^; however, studies consistently reported that food provided in ECEC exceeded the Caroline Walker Trust (CWT) and ‘Eat Better, Start Better’ (EBSB) recommendations for fat and salt and was deficient in fibre, Fe and Zn^(48,63)^. Frequent provision of cakes and biscuits was identified as contributing to excess sugar provided in ECEC^(63)^.

Two studies reported that portion sizes served in school-based nurseries were more compliant with school food standards than ECEC recommendations^(63,64)^. Another study reported that settings in more deprived areas had better nutrition index ratings^(47)^.

#### Barriers to healthy food provision

Findings from the included studies highlighted several barriers to providing healthy foods faced by ECEC including financial constraints, time capacity, type of ECEC and source of food. Three studies identified that time constraints impacted what food was provided in ECEC^(50,53,54)^. Childminders felt strongly that their limited time capacity negatively impacted food provision and resulted in a reliance on processed convenience foods^(53)^. Similarly, another study reported that the quality of ingredients used for food preparation in ECEC was largely governed by financial and time capacity^(54)^.

The type of ECEC was also a factor that impacted food provision. School-based nurseries had little to no control over what food could be purchased, as the budget for consumables was controlled by management in the associated primary school^(54)^. In addition, parents held more power over menu choices in private nurseries, as they were considered customers to a ‘business’^(49)^. Consequently, private nurseries in less deprived areas were able to provide good quality foods, as parents had greater financial responsibility^(50)^. Nurseries that prepared food on site with a dedicated cook had more autonomy over menu development than settings that relied on an external catering company^(49)^. Additionally, nurseries with the farthest distance to the nearest supermarket were more likely to serve fruit and vegetables less than 2–3 times per week^(61)^.

#### Recommendations for food provision-related policy development and future research

The authors of the studies had minimal recommendations for policy development related to food provision in ECEC. One study recommended that there should be increased support for schools and caterers providing food for multiple age groups to ensure that appropriate portion sizes and nutrient requirements are served to the respective age group^(63)^.

Moore et al. (2005) identified a need for future research to explore the nutritional quality of food served in ECEC^(52)^. After concluding that offering vegetables to children at breakfast time was feasible, one study recommended that a future randomised controlled trial should be undertaken to explore this intervention further^(42)^.

## Discussion

This is the first review of studies to explore food policies, feeding practices and food provision in UK ECEC. We used the SEM to synthesise key findings and recommendations for further research. Twenty-four studies were identified, and the majority used qualitative methods or were of cross-sectional design. Most of the studies were conducted in England in a nursery setting and included ECEC staff as participants, with no studies undertaken in Northern Ireland or Wales. Studies most frequently focused on food practices in ECEC, followed by provision and then policy. The majority of research was at the organisational SEM level, and very few studies targeted governmental and individual levels.

The focus on UK countries was important to clearly see the scope of research within the UK and compare to international studies. Reviews of studies undertaken internationally have focused on synthesising ECEC nutrition interventions^(65–71)^, whereas our review identified only three studies that used experimental methods, indicating a stark difference in the type of evidence that characterises the UK ECEC research landscape. Despite differences in study design, international literature reviews generally reported that barriers to food policy implementation, healthy feeding practices and nutritional quality in ECEC were consistent with those identified from UK literature^(68,72)^. Ultimately, the gaps in research and policy highlighted in international literature reviews support our recommendations for future research that targets all five SEM levels^(68–70)^ and for increased food-related ECEC policies^(67)^.

### Policy

Our findings suggested that food policies and the way they are implemented are likely to be highly variable across ECEC in the UK, with differences most evident between private and local authority-funded settings. A recent report by Nourishing Our Future found that 81 % of settings in Essex, England, have a food policy, which is higher than findings reported within literature and further highlights the wide variability in food policy use across the UK^(73)^. The variability in the content of ECEC food policies is expected, as ECEC are advised to adapt policies to reflect the individual setting^(74)^. However, providing a more specific policy framework and support for policy development, such as those provided by Australian States/Territories to ECEC, would be beneficial for UK ECEC to develop more specific and comprehensive food policies^(67)^. Furthermore, it was evident that ECEC struggled to fully adhere to food policies once they were in place. Previous evidence from settings based in Australia has shown that implementation and adherence to food policies in ECEC could be improved with active support from ECEC managers, parents and accessible resources^(75)^.

This review found that there was poor implementation of government schemes in UK ECEC^(44,55)^. Government schemes have been effective in the school environment. The School Food Standards and Universal Infant Free School Meals (UIFSM) have improved dietary intake at lunchtimes, with particularly beneficial impacts for low-income children^(76,77)^. Similarly, government legislation in Sweden entitles all children in preschool and primary school to a free and nutritious meal, which has been effective in ensuring school meal quality and beneficial feeding practices^(78,79)^. The evident success of government schemes in the school environment and in international settings suggests that similar schemes could be beneficial for food quality and feeding practices in UK ECEC if implemented universally, and with sufficient support and resources for ECEC staff ^(67)^.

### Practice

This review found that staff role modelling was a common practice reported in UK ECEC. Role modelling is a widely recommended practice to promote healthy eating and young children’s acceptance of unfamiliar foods^(80)^. Our review also found that it was common practice to allow children to self-serve, which evidence suggests is beneficial for reducing energy intake^(81)^. Similarly, the involvement of children in food preparation has positive impacts on vegetable intake in children^(82)^. Whilst this practice is common within ECEC in countries such as New Zealand, it was not frequently reported in UK ECEC^(83)^. Diluting fruit juice with water was a practice reported in more deprived ECEC. This practice reflected previous recommendations that have since been revised; current guidance recommends only water or milk in ECEC^(84,85)^. This was an important practice given the high proportion of sugar found in juice drinks marketed to children and subsequently high intake of sugar from sugar-sweetened beverages in UK children^(86,87)^.

Although this review found that feeding practices were generally positive, providers also displayed practices that were not recommended such as using ‘treats’ as rewards for good behaviour or expecting children to finish their meals. The use of ‘treats’ such as sweets or desserts as rewards during childhood has associations with dietary behaviours later in life, as well as negative outcomes related to physical and mental health^(88,89)^. Similarly, we found that some settings in more deprived areas expected children to finish all their meals^(59)^, which is not encouraged as this can affect awareness of hunger and fullness cues, important for self-regulation of energy intake^(90)^. However, children in deprived areas are more likely to face food insecurity, and therefore staff may want to ensure children have had sufficient intake during their care^(91)^. Our review also found that ECEC staff tended to cite sources of information that were not official healthy eating guidance, such as advice from parents or childminding magazines^(49)^. Previous evidence has shown that parents of early years children are also generally unaware of portion size guidance, indicating that ECEC staff should not be reliant on parents for feeding practice advice^(92)^. Findings from the Nourishing Our Future report identified that settings found guidance resources too complex and overwhelming, or not visual enough, which is consistent with findings from this review^(73)^. This therefore highlights a need for intervention to promote effective use and awareness of nutrition guidance resources.

Notably, the majority of findings related to feeding practices in UK ECEC came from qualitative interviews or self-reported questionnaires by providers. A previous study found that there were disparities between reported practices by caregivers and actual practices observed by researchers^(93)^. There is therefore a need for improved methodology to accurately capture feeding practices in the ECEC environment.

### Provision

On average, the lunches provided in UK ECEC had excess fat, sugars, protein and salt and were deficient in Fe and Zn^(48,63)^. These findings are similar to those found in primary schools in England before the introduction of The School Food Standards in 2008, where children consumed excess fat and deficient amounts of energy, carbohydrates and Fe from school lunches^(94)^. Additionally, the excess energy and protein and inadequate Fe intake is a widespread observation across European countries for early years children and in ECEC^(95,96)^. We identified cost as a barrier to healthy food provision in UK ECEC, which is an unsurprising consequence of the chronic underfunding faced by ECEC in the UK^(30,97)^. This was most evident in settings in more deprived areas, which have been disproportionately affected by funding changes^(41,98)^.

Our findings also showed that school-based nurseries served inappropriately large portion sizes that were more suitable for primary school-aged recommendations than early years^(63,64)^. These findings could partly explain why a previous study found that eating occasions were larger in childcare than in the home setting^(21)^. The consumption of such large portion sizes could have negative health implications for early years such as an increased risk of high body weight and blood pressure due to excess energy and salt intake^(99,100)^. It could be postulated that the inappropriate portion sizes in ECEC could be a result of lack of training and nutrition education that was reported in ECEC staff and cooks, or the limited awareness and use of ECEC-specific feeding guidance.

### The effect of deprivation

This review found that ECEC in the most deprived areas adhered to more recommended feeding practices and provided more nutritional foods than settings in less deprived areas. For example, one study reported that settings in the most deprived areas were more likely to dilute juice with water ^(59)^. Whilst diluting juice is typically a practice to reduce sugar consumption, it is more likely that ECEC in deprived areas comply with this practice to reduce costs as ECEC may face significant budgetary constraints in more deprived areas^(30)^. Alternatively, these findings could reflect the differences found between ECEC types, as children from more deprived areas are more likely to attend local authority-funded settings^(101)^, which have more support to assist food policy development and healthy feeding practices.

Our findings contrast a previous study that found greater socio-economic deprivation was associated with poorer nutritional quality of food in English secondary schools^(102)^. More generally, it has been established that lower household income and socio-economic deprivation are associated with poorer diet quality in children^(103)^. The findings in this review therefore present promising evidence that ECEC could act as key mitigators to improve dietary intake of children in deprived areas and indicate that further research is needed to explore food provision and practice in ECEC that face deprivation.

### Implications

This scoping review found that the majority of research on food policy, practice and provision in UK ECEC was at the organisational SEM level. This is problematic as it continues to place the responsibility on providers that are typically overstretched and underfunded^(30)^. Future research should therefore aim to develop and evaluate governmental programmes and policies to ensure that they are effective at supporting and improving child health. Given the success of previous interventions that have targeted ECEC in other countries^(105–107)^, there is also scope for more healthy eating interventions in UK ECEC. Furthermore, interventions in ECEC may be more impactful if they use a multi-level approach, targeting a range of SEM levels. Our findings highlighted a need for quantitative research that more accurately captures the nutritional quality of food provided and consumed, as well as further exploring the impact of cost on food provision in the current economic climate.

The most striking finding from this review is the urgent need for research in Wales, Northern Ireland and Scotland. The ongoing National Institute of Health and Care Research (NIHR) ‘Growing Well Study’ (GWS)^(108)^, whilst important to explore food and nutrient intake in English preschool children, further perpetuates this lack of research conducted in the three other UK countries. Similarly, more research is needed that represents a range of deprivation levels to effectively identify barriers faced by ECEC across the UK.

The findings from this review provide an evidence base to support policy change needed in UK ECEC. Our findings indicate that the introduction of the statutory nutrition requirements in English ECEC from September 2025 will be beneficial for ensuring adequate nutritional quality in food provided to early years children^(22)^. Similar nutrition requirements should also be in place for ECEC in Wales and Northern Ireland. The Early Years Foundation Stage (EYFS) nutrition guidance was published in May 2025 to support ECEC with the new statutory nutrition requirements. This provides more accessible and succinct advice for ECEC on food groups, food policy development and menu planning than previous guidance. However, the EYFS guidance lacks clarity on portion sizes and feeding practices, which we have established as areas requiring further support for ECEC^(74)^. Increased governmental support for the implementation of nutrition requirements is important to ensure additional burden is not placed on ECEC providers.

Additionally, our findings indicated that nutrition education and practice training should be made more accessible to ECEC staff through government-funded schemes and should be monitored by Ofsted to ensure recommended feeding practices are upheld. Finally, the findings from this review, coupled with the success of UIFSM scheme, support the proposition for universal-free meals in ECEC, which would alleviate cost as a barrier to nutritional food provision and help reduce inequality between ECEC types and deprivation levels.

### Strengths and limitations

Strengths of this scoping review include the comprehensive and systematic search of peer-reviewed literature and inclusion of a breadth of study designs, thereby ensuring all relevant, available evidence has been collated to scope what is known about the food policy, provision and practice in UK ECEC. Using a socio-ecological approach was also a strength of this review, as it informed where gaps in research lie and highlighted how a multi-level approach for future studies would provide impactful insights on food policy, practice and provision in ECEC.

There were also a number of limitations to this review. For example, this scoping review did not include grey literature which may have provided further context of food provision and practices in ECEC, as well as evidence that supports the need for policy changes. However, this review can be used alongside a published grey literature review of early years portion size guidance resources in the UK and Ireland^(109)^ and grey literature reports from early years advocacy organisations to call for policy change. Another limitation of this review is that five of the studies were published before 2012^(44,47,48,50,52)^, which predates the publication of CWT, EBSB and EYFS guidelines^(74,84,110)^ and therefore the studies do not evaluate food practices and provision against these current ECEC guidelines. There have also been recent geopolitical and economic changes since many of the studies were published, and therefore findings may not reflect current food policy, practices or provision in UK ECEC.

### Conclusion

Overall, we found that UK ECEC had generally poor adherence to food policies, and government schemes were not implemented effectively in the ECEC environment. Although feeding practices reported were mostly positive, a lack of nutrition training and awareness of guidance was apparent in ECEC staff. Barriers to healthy food provision included financial constraints, time capacity, type of ECEC and source of food. Our findings show that there is an urgent need for an increased focus on research and policy addressing the food environment in UK ECEC, specifically in Scotland, Northern Ireland and Wales. Future research should aim to capture a range of influences affecting food policy, practice and provision in UK ECEC to ensure that responsibility is not placed solely on providers and to inform future policy development.

## Supporting information

Turner et al. supplementary material 1 Turner et al. supplementary material

Turner et al. supplementary material 2 Turner et al. supplementary material

## Supplementary material

For supplementary material accompanying this paper visit https://doi.org/10.1017/S1368980025101298

## References

[1] The Lancet Public Health (2025) Time to tackle obesogenic environments. Lancet Public Health 10, e165. 40044241 10.1016/S2468-2667(25)00049-0

[2] NHS England (2024) National Child Measurement Programme, England, 2023/24 School Year. https://digital.nhs.uk/data-and-information/publications/statistical/national-child-measurement-programme/2023-24-school-year (accessed 08 April 2025).

[3] Cabinet Secretary for Health and Social Care (2024) The Scottish Health Survey 2023. https://www.gov.scot/publications/scottish-health-survey-2023-volume-1-main-report/documents/ (accessed 08 April 2025).

[4] The Department of Health (2020) Health Survey (NI): First Results 2019/20. https://www.health-ni.gov.uk/news/health-survey-ni-first-results-201920#:~:text=Whilst%20obesity%20levels%20were%20similar%2C%20males%20were%20more,as%20overweight%20and%206%25%20were%20classed%20as%20obese (accessed 08 April 2025).

[5] Public Health Wales NHS Trust (2024) Child Measurement Programme 2022/23. https://phw.nhs.wales/services-and-teams/child-measurement-programme/cmp-2022-23/1-cmp-report-2022-2023/ (accessed 08 April 2025).

[6] Department of Health & Social Care (2020) Tackling Obesity: Empowering Adults and Children to Live Healthier Lives. https://www.gov.uk/government/publications/tackling-obesity-government-strategy/takling-obesity-empowering-adults-and%20children-to-live-healthier-lives (accessed 08 April 2025).

[7] Scottish Government (2018) A Healthier Future: Scotland’s Diet and Healthy Weight Delivery Plan. https://www.gov.scot/publications/healthier-future-scotlands-diet-healthy-weight-delivery-plan/ (accessed 08 April 2025).

[8] Welsh Government (2023) Healthy Weight Strategy *Healthy Weight Healthy Wales*. https://www.gov.wales/healthy-weight-strategy-healthy-weight-healthy-wales (accessed 08 April 2025).

[9] Liberali R , Kupek E & Assis MA (2019) Dietary patterns and childhood obesity risk: a systematic review. Childhood Obes 16, 70–85. 10.1089/chi.2019.0059 31742427

[10] Ambrosini GL , Emmett PM , Northstone K et al. (2012) Identification of a dietary pattern prospectively associated with increased adiposity during childhood and adolescence. Int J Obes 36, 1299–1305. 10.1038/ijo.2012.127 PMC3466487 22868831

[11] Balthazar EA & de Oliveira MR (2011) Differences in dietary pattern between obese and eutrophic children. BMC Res Notes 4, 567. 22206728 10.1186/1756-0500-4-567 PMC3339399

[12] Magriplis E , Farajian P , Panagiotakos DB et al. (2019) The relationship between behavioral factors, weight status and a dietary pattern in primary school aged children: the GRECO study. Clin Nutr 38, 310–316. 29398340 10.1016/j.clnu.2018.01.015

[13] Maynard M , Gunnell D , Emmett P et al. (2003) Fruit, vegetables, and antioxidants in childhood and risk of adult cancer: the Boyd Orr cohort. J Epidemiol Community Health 57, 218–225. 12594199 10.1136/jech.57.3.218 PMC1732406

[14] Ness AR , Maynard M , Frankel S et al. (2005) Diet in childhood and adult cardiovascular and all cause mortality: the Boyd Orr cohort. Heart 91, 894–898. 15958357 10.1136/hrt.2004.043489 PMC1768996

[15] Smithers LG , Golley RK , Mittinty MN et al. (2012) Dietary patterns at 6, 15 and 24 months of age are associated with IQ at 8 years of age. *Eur J Epidemiol* **27**, 525–535. 10.1007/s10654-012-9715-5 22810299

[16] Tandon PS , Tovar A , Jayasuriya AT et al. (2016) The relationship between physical activity and diet and young children’s cognitive development: a systematic review. Prev Med Rep 3, 379–390. 27419040 10.1016/j.pmedr.2016.04.003 PMC4929214

[17] Evans CEL , Hutchinson J , Christian MS et al. (2018) Measures of low food variety and poor dietary quality in a cross-sectional study of London school children. Eur J Clin Nutr 72, 1497–1505. 29391590 10.1038/s41430-017-0070-1

[18] Public Health England UFSA (2021) *National Diet and Nutrition Survey: Diet, Nutrition and Physical Activity in 2020*. National Diet and Nutrition Survey no. GOV-9714.

[19] Scottish Government (2020) Scottish Household Survey: Childcare Topic Report. https://www.gov.scot/publications/scottish-household-survey-childcare-topic-report/pages/2/ (accessed 08 April 2025).

[20] Department for Education (2024) Childcare and Early Years Survey of Parents. https://explore-education-statistics.service.gov.uk/find-statistics/childcare-and-early-years-survey-of-parents/2023#dataBlock-f496b072-cf84-4911-a395-c1c78bd09a76-tables (accessed 08 April 2025).

[21] Porter A , Toumpakari Z , Kipping R et al. (2021) Where and when are portion sizes larger in young children? An analysis of eating occasion size among 1·5–5-year-olds in the UK National Diet and Nutrition Survey (2008–2017). Public Health Nutr 25, 1–12. 10.1017/S1368980021005024 PMC9991682 34955105

[22] UK Government Childcare Act 2016, c. 5. (2022) https://www.legislation.gov.uk/ukpga/2016/5/section/1/2022-03-25 (accessed 08 April 2025).

[23] Department for Education (n.d) Tax-Free Childcare. https://www.gov.uk/tax-free-childcare (accessed 08 April 2025).

[24] Department for Education (2024) Executive Early Learning and Childcare Strategy. https://www.education-ni.gov.uk/articles/executive-early-learning-and-childcare-strategy#toc-0 (accessed 08 April 2025).

[25] Wainwright D , BBC Verify & Clarke V (2023) Childcare: Mum-to-be Given Two-Year Wait as Demand Rises. https://www.bbc.co.uk/news/education-67274943 (accessed 08 April 2025).

[26] Department for Education (2024) September 2025 Early Education and Childcare Entitlements Expansion. https://assets.publishing.service.gov.uk/media/67056cf7366f494ab2e7b52b/September_25_early_years_entitlements_expansion_system_guidance.pdf (accessed 29 April 2025).

[27] UK Government (n.d) Get Free Childcare If You’re Working. https://www.gov.uk/free-childcare-if-working/what-youll-get (accessed 08 April 2025).

[28] UK Government (2024) Education (Scotland) Act 1980 c. 44.

[29] Molcho M , Gabhainn SN , Kelly C et al. (2007) Food poverty and health among schoolchildren in Ireland: findings from the Health Behaviour in School-aged Children (HBSC) study. Public Health Nutr 10, 364–370. 17362532 10.1017/S1368980007226072

[30] National Day Nurseries Association (2021) Stop Underfunding- Start Building Futures England. https://ndna.org.uk/wp-content/uploads/2021/09/1258_NDNA_A4_Report_PDF_England_AW-1.pdf (accessed 08 April 2025).

[31] Procter KL , Clarke GP , Ransley JK et al. (2008) Micro-level analysis of childhood obesity, diet, physical activity, residential socioeconomic and social capital variables: where are the obesogenic environments in Leeds? *Area* **40**, 323–340.

[32] Brofenbrenner U (1977) Toward an experimental ecology of human development. Am Psychol Assoc 32, 513–531.

[33] Ohri-Vachaspati P , DeLia D , DeWeese RS et al. (2015) The relative contribution of layers of the Social Ecological Model to childhood obesity. Public Health Nutr 18, 2055–2066. 25374257 10.1017/S1368980014002365 PMC4775271

[34] Pereira M , Padez C & Nogueira H (2019) Describing studies on childhood obesity determinants by Socio-Ecological Model level: a scoping review to identify gaps and provide guidance for future research. Int J Obes 43, 1883–1890. 10.1038/s41366-019-0411-3 31285521

[35] Aromataris E , Lockwood C , Porritt K et al. (2024) *JBI Manual for Evidence Synthesis*. Adelaide, SA: JBI.

[36] McGowan J , Straus S , Moher D et al. (2020) Reporting scoping reviews-PRISMA ScR extension. J Clin Epidemiol 123, 177–179. 32229248 10.1016/j.jclinepi.2020.03.016

[37] Tricco AC , Lillie E , Zarin W et al. (2018) PRISMA extension for scoping reviews (PRISMA-ScR): checklist and explanation. Ann Intern Med 169, 467–473. 30178033 10.7326/M18-0850

[38] Chinn S & Rona RJ (2001) Prevalence and trends in overweight and obesity in three cross sectional studies of British Children, 1974–94. BMJ 322, 24–26. 11141148 10.1136/bmj.322.7277.24 PMC26603

[39] Ouzzani M , Hammady H , Fedorowicz Z et al. (2016) Rayyan-a web and mobile app for systematic reviews. Syst Rev 5, 210. 27919275 10.1186/s13643-016-0384-4 PMC5139140

[40] Taylor-Phillips S , Geppert J , Stinton C et al. (2017) Comparison of a full systematic review *v.* rapid review approaches to assess a newborn screening test for tyrosinemia type 1. Res Synth Methods 8, 475–484. 28703492 10.1002/jrsm.1255

[41] Langford R , Jago R , White J et al. (2019) A physical activity, nutrition and oral health intervention in nursery settings: process evaluation of the NAP SACC UK feasibility cluster RCT. BMC Public Health 19, 865–865. 31269926 10.1186/s12889-019-7102-9 PMC6609387

[42] McLeod CJ , Haycraft E & Daley AJ (2023) Offering vegetables to children at breakfast time in nursery and kindergarten settings: the Veggie Brek feasibility and acceptability cluster randomised controlled trial. *Int J Behav Nutr Phys Act* **20**, ArtID 38. 10.1186/s12966-023-01443-z PMC10043832 36978097

[43] Nekitsing C , Blundell-Birtill P , Cockroft JE et al. (2019) Taste exposure increases intake and nutrition education increases willingness to try an unfamiliar vegetable in preschool children: a cluster randomized trial. J Academy Nutr Diet 119, 2004–2013. 10.1016/j.jand.2019.05.012 31378647

[44] Albon D (2009) Challenges to improving the uptake of milk in a nursery class: a case study. Health Educ 109, 140–154.

[45] Er V , Dias KI , Papadaki A et al. (2018) Association of diet in nurseries and physical activity with zBMI in 2–4-year olds in England: a cross-sectional study. BMC Public Health 18, 1262. 30428858 10.1186/s12889-018-6138-6 PMC6236905

[46] Marr C , Breeze P & Caton SJ (2022) Examination of dietary intake of UK preschool children by varying carers: evidence from the 2008–2016 UK National Diet and Nutrition Survey. Br J Nutr 128, 2063–2074. 34842127 10.1017/S0007114521004712

[47] Alderton T & Campbell-Barr V (2005) Quality early education–quality food and nutrition practices? Some initial results from a pilot research project into food and nutrition practices in early years settings in Kent, UK. Int J Early Years Educ 13, 197–213.

[48] Parker M , Lloyd-Williams F , Weston G et al. (2011) Nursery nutrition in Liverpool: an exploration of practice and nutritional analysis of food provided. Public Health Nutr 14, 1867–1875. 21729488 10.1017/S1368980011000887

[49] Buttivant H & Knai C (2012) Improving food provision in child care in England: a stakeholder analysis. Public Health Nutr 15, 554–560. 21859504 10.1017/S1368980011001704

[50] Lloyd-Williams F , Bristow K , Capewell S et al. (2011) Young children’s food in Liverpool day-care settings: a qualitative study of pre-school nutrition policy and practice. Public Health Nutr 14, 1858–1866. 21557874 10.1017/S1368980011000619

[51] McSweeney LA , Rapley T , Summerbell CD et al. (2016) Perceptions of nursery staff and parent views of healthy eating promotion in preschool settings: an exploratory qualitative study. BMC Public Health 16, 841. 27542605 10.1186/s12889-016-3507-x PMC4992270

[52] Moore H , Nelson P , Marshall J et al. (2005) Laying foundations for health: food provision for under 5s in day care. Appetite 44, 207–213. 15808895 10.1016/j.appet.2004.08.009

[53] Goldsborough N , Homer C , Atchinson R et al. (2016) Healthy eating in the early years: a qualitative exploration of food provision in the childminder setting. Br Food J 118, 992–1002.

[54] Bristow K , Povall S , Capewell S et al. (2013) Exploring health inequalities through the lens of an ethnographic study of healthy eating provision in the early years sector. Matern Child Nutr 9, 260–273. 22118155 10.1111/j.1740-8709.2011.00359.x PMC6860518

[55] Warren E , Williams L & Knai C (2022) The ‘Cinderella sector’: the challenges of promoting food and nutrition for young children in early years’ settings in England. Ecol Food Nutr 61, 576–594. 35579381 10.1080/03670244.2022.2073353

[56] Williams L , Warren E & Knai C (2022) How involved are parents in their child’s early years setting’s food decisions and practices? *SSM Qual Res Health* **2**, 100142. 10.1016/j.ssmqr.2022.100142 PMC9748307 36606099

[57] Quirke-McFarlane S , Carstairs SA & Cecil JE (2024) ‘You just eyeball it’: parent and nursery staff perceptions and influences on child portion size: a reflexive thematic analysis. Nutr Health 31, 701–714. 38623628 10.1177/02601060241245255 PMC12174632

[58] Elford L & Brown A (2014) Exploring child-feeding style in childcare settings: how might nursery practitioners affect child eating style and weight? Eating Behav 15, 314–317. 10.1016/j.eatbeh.2014.04.001 24854825

[59] Neelon SE , Burgoine T , Hesketh KR et al. (2015) Nutrition practices of nurseries in England. Comparison with national guidelines. Appetite 85, 22–29. 25450898 10.1016/j.appet.2014.11.002 PMC4286113

[60] Warren E , Boadu P , Exley J et al. (2024) Knowledge and use of voluntary food and drink guidelines in English nurseries? Results from a nationally representative cross-sectional study. Food Policy 122, 102573.

[61] Burgoine T , Gallis JA , Penney TL et al. (2017) Association between distance to nearest supermarket and provision of fruits and vegetables in English nurseries. Health Place 46, 229–233. 28595138 10.1016/j.healthplace.2017.05.018 PMC5537193

[62] NHS (2022) Fish and Shellfish. https://www.nhs.uk/live-well/eat-well/food-types/fish-and-shellfish-nutrition/ (accessed 30 April 2025).

[63] Wall CJ & Pearce J (2023) Energy and nutrient content of school lunches provided for children attending school-based nurseries: a cross-sectional study. Public Health Nutr 26, 2641–2651. 37921199 10.1017/S1368980023002331 PMC10755416

[64] Pearce J & Wall CJ (2023) School lunch portion sizes provided for children attending early years settings within primary schools: a cross-sectional study. J Hum Nutr Diet 36, 1887–1900. 37278164 10.1111/jhn.13183

[65] Bell LK & Golley RK (2015) Interventions for improving young children’s dietary intake through early childhood settings: a systematic review. Int J Child Health Nutr 4, 14–32.

[66] Chan J , Conroy P , Phongsavan P et al. (2023) Systems map of interventions to improve dietary intake of pre-school aged children: a scoping review. Prev Med 177, 107727. 37848165 10.1016/j.ypmed.2023.107727

[67] Lucas PJ , Patterson E , Sacks G et al. (2017) Preschool and school meal policies: an overview of what we know about regulation, implementation, and impact on diet in the UK, Sweden, and Australia. Nutrients 9, 736. 28696403 10.3390/nu9070736 PMC5537850

[68] Matwiejczyk L , Mehta K , Scott J et al. (2018) Characteristics of effective interventions promoting healthy eating for pre-schoolers in childcare settings: an umbrella review. Nutrients 10, 293. 29494537 10.3390/nu10030293 PMC5872711

[69] Mikkelsen MV , Husby S , Skov LR et al. (2014) A systematic review of types of healthy eating interventions in preschools. Nutr J 13, 56. 24906305 10.1186/1475-2891-13-56 PMC4074866

[70] Wolfenden L , Jones J , Williams CM et al. (2016) Strategies to improve the implementation of healthy eating, physical activity and obesity prevention policies, practices or programmes within childcare services. *Cochrane Database Syst Rev* issue 10, CD011779. 10.1002/14651858.CD011779.pub2 PMC6458009 27699761

[71] Yoong SL , Lum M , Wolfenden L et al. (2023) Healthy eating interventions delivered in early childhood education and care settings for improving the diet of children aged six months to six years. *Cochrane Database Syst Rev* issue 6, CD013862. 10.1002/14651858.CD013862.pub2 PMC10259732 37306513

[72] Willemsen A , Wiggins S & Cromdal J (2023) Young children’s mealtimes and eating practices in early childhood education and care: a scoping review of 30 years of research from 1990 to 2020. Educ Res Rev 38, 100503.

[73] Nourishing Our Future (2025) Exploring Food Provision in Essex Early Years Settings. https://nourishingourfuture.co.uk/wp-content/uploads/2025/03/digital-nof-conference-report-march2025-1.pdf (accessed 18 August 2025).

[74] Department for Education (2025) Early Years Foundation Stage Nutrition Guidance. https://assets.publishing.service.gov.uk/media/67f8e61c04146682e61bc84c/Nutrition_guidance_for_early_years_providers.pdf (accessed 30 April 2025).

[75] Wolfenden L , Finch M , Nathan N et al. (2015) Factors associated with early childhood education and care service implementation of healthy eating and physical activity policies and practices in Australia: a cross-sectional study. Translational Behav Med 5, 327–334. 10.1007/s13142-015-0319-y PMC4537457 26327938

[76] Adamson A , Spence S , Reed L et al. (2013) School food standards in the UK: implementation and evaluation. Public Health Nutr 16, 968–981. 23578662 10.1017/S1368980013000621 PMC10271333

[77] Parnham JC , Chang K , Millett C et al. (2022) The impact of the universal infant free school meal policy on dietary quality in English and Scottish primary school children: evaluation of a natural experiment. Nutrients 14, 1602. 35458164 10.3390/nu14081602 PMC9029848

[78] Patterson E & Elinder LS (2015) Improvements in school meal quality in Sweden after the introduction of new legislation-a 2-year follow-up. Eur J Public Health 25, 655–660. 25395403 10.1093/eurpub/cku184

[79] Persson Osowski C , Göranzon H & Fjellström C (2013) Teachers’ interaction with children in the school meal situation: the example of pedagogic meals in Sweden. J Nutr Educ Behav 45, 420–427. 23768894 10.1016/j.jneb.2013.02.008

[80] Harper LV & Sanders KM (1975) The effect of adults’ eating on young children’s acceptance of unfamiliar foods. J Exp Child Psychol 20, 206–214.

[81] Fisher JO , Rolls BJ & Birch LL (2003) Children’s bite size and intake of an entrée are greater with large portions than with age-appropriate or self-selected portions. Am J Clin Nutr 77, 1164–1170. 12716667 10.1093/ajcn/77.5.1164 PMC2530925

[82] van der Horst K , Ferrage A & Rytz A (2014) Involving children in meal preparation. Effects on food intake. Appetite 79, 18–24. 24709485 10.1016/j.appet.2014.03.030

[83] Gerritsen S , Wall C & Morton S (2016) Child-care nutrition environments: results from a survey of policy and practice in New Zealand early childhood education services. Public Health Nutr 19, 1531–1542. 26466671 10.1017/S1368980015002955 PMC10271085

[84] Action for Children (2017) Eat Better, Start Better Voluntary Food and Drink Guidelines for Early Years Settings in England. https://foundationyears.org.uk/files/2017/11/Eat-Better-Start-Better1.pdf (accessed 08 April 2025).

[85] Children’s Food Trust (2012) Eat Better Start Better Voluntary Food and Drink Guidelines for Early Years Settings in England – A Practical Guide. https://miniminersnursery.co.uk/eat-better-start-better-short-version.pdf (accessed 29 August 2025).

[86] Boulton J , Hashem KM , Jenner KH et al. (2016) How much sugar is hidden in drinks marketed to children? A survey of fruit juices, juice drinks and smoothies. BMJ Open 6, e010330. 10.1136/bmjopen-2015-010330 PMC4809069 27009146

[87] Seferidi P , Millett C & Laverty AA (2018) Sweetened beverage intake in association to energy and sugar consumption and cardiometabolic markers in children. Pediatr Obes 13, 195–203. 28112866 10.1111/ijpo.12194

[88] Fedewa AL & Davis MC (2015) How food as a reward is detrimental to children’s health, learning, and behavior. J Sch Health 85, 648–658. 26201761 10.1111/josh.12294

[89] Puhl RM & Schwartz MB (2003) If you are good you can have a cookie: how memories of childhood food rules link to adult eating behaviors. Eating Behav 4, 283–293. 10.1016/S1471-0153(03)00024-2 15000971

[90] Johnson SL (2000) Improving preschoolers’ self-regulation of energy intake. Pediatrics 106, 1429–1435. 11099599 10.1542/peds.106.6.1429

[91] Stanley AK , Hadi Y , Newbold D et al. (2025) Identifying predictors for food insecurity in England: a cross-sectional database analysis. J Health Popul Nutr 44, 53. 40022274 10.1186/s41043-025-00801-w PMC11871808

[92] Porter A , Langford R , Summerbell C et al. (2023) A qualitative exploration of food portion size practices and awareness of food portion size guidance in first-time parents of one- to two-year-olds living in the UK. BMC Public Health 23, 1779. 37704981 10.1186/s12889-023-16647-y PMC10500748

[93] Erinosho TO , Hales DP , McWilliams CP et al. (2012) Nutrition policies at child-care centers and impact on role modeling of healthy eating behaviors of caregivers. J Academy Nutr Diet 112, 119–124. 10.1016/j.jada.2011.08.048 22709641

[94] Rogers IS , Ness AR , Hebditch K et al. (2007) Quality of food eaten in English primary schools: school dinners *v.* packed lunches. Eur J Clin Nutr 61, 856–864. 17213869 10.1038/sj.ejcn.1602592

[95] EFSA Panel on Dietetic Products N & Allergies (2013) Scientific opinion on nutrient requirements and dietary intakes of infants and young children in the European Union. EFSA J 11, 3408.

[96] Goldbohm RA , Rubingh CM , Lanting CI et al. (2016) Food consumption and nutrient intake by children aged 10 to 48 months attending day care in The Netherlands. Nutrients 8, 428. 27428995 10.3390/nu8070428 PMC4963904

[97] Lawler R (2021) New Data Shows Ministers Knew Early Years was Underfunded. https://www.eyalliance.org.uk/news/2021/06/new-data-shows-ministers-knew-early-years-was-underfunded (accessed 08 April 2025).

[98] Stewart K , Gambaro L & Reader M (2025) Levelling down? Understanding the decline of the maintained nursery sector in England. *Br Educ Res J* **51**, 1009–1038.

[99] He FJ , Marrero NM & MacGregor GA (2008) Salt and blood pressure in children and adolescents. J Hum Hypertens 22, 4–11. 17823599 10.1038/sj.jhh.1002268

[100] Swinburn BA , Jolley D , Kremer PJ et al. (2006) Estimating the effects of energy imbalance on changes in body weight in children. Am J Clin Nutr 83, 859–863. 16600939 10.1093/ajcn/83.4.859

[101] Gambaro L , Stewart K , Waldfogel J et al. (2014) Equal access to early childhood education and care? The case of the UK. In *An Equal Start?: Providing Quality Early Education and Care for Disadvantaged Children*, pp. 29–52 [ L Gambaro , K Stewart , J Waldfogel , editors]. Bristol: Policy Press Scholarship Online.

[102] Gould R , Russell J & Barker ME (2006) School lunch menus and 11 to 12 year old children’s food choice in three secondary schools in England—are the nutritional standards being met? Appetite 46, 86–92. 16298457 10.1016/j.appet.2005.08.005

[103] Nelson M (2000) Childhood nutrition and poverty. *Proc Nutr Soc* **59**, 307–315. 10.1017/s0029665100000343 10946800

[104] Dombrowski L , Henderson S , Leslie J et al. (2020) The role of early years care providers in supporting continued breastfeeding and breast milk feeding. Early Years: Int J Res Dev 40, 205–220.

[105] Davis SM , Sanders SG , FitzGerald CA et al. (2013) CHILE: an evidence-based preschool intervention for obesity prevention in head start. J Sch Health 83, 223–229. 23343323 10.1111/josh.12018 PMC3556909

[106] Gans KM , Tovar A , Kang A et al. (2022) A multi-component tailored intervention in family childcare homes improves diet quality and sedentary behavior of preschool children compared to an attention control: results from the Healthy Start-Comienzos Sanos cluster randomized trial. Int J Behav Nutr Phys Act 19, 45. 35428298 10.1186/s12966-022-01272-6 PMC9013065

[107] Puder JJ , Marques-Vidal P , Schindler C et al. (2011) Effect of multidimensional lifestyle intervention on fitness and adiposity in predominantly migrant preschool children (Ballabeina): cluster randomised controlled trial. BMJ 343, d6195. 21998346 10.1136/bmj.d6195 PMC3192456

[108] Cade JE , Threapleton D , Greenwood D et al. (2025) Nutrition in Pre-School Children (NIPre) Study: The Role of Portion Size, Plant-Based Foods and Commercially Manufactured Foods and Drinks. https://fundingawards.nihr.ac.uk/award/NIHR207073 (accessed 08 April 2025).

[109] Porter A , Kipping R , Summerbell C et al. (2020) What guidance is there on portion size for feeding preschool-aged children (1 to 5 years) in the United Kingdom and Ireland? A systematic grey literature review. Obes Rev 21, e13021. 32219990 10.1111/obr.13021

[110] The Caroline Walker Trust (2011) *Eating Well for 1–4 Year Olds Practical Guide*, 2nd ed. Hertfordshire: The Caroline Walker Trust.

