# Supplementary material for: Food policy, practice and provision in UK early childhood education and care: a scoping review
Source: Public Health Nutr. 2025 Oct 13;28(1):e176. doi: 10.1017/S1368980025101298 (PMC12722074; doi:10.1017/S1368980025101298)
Supplement: Turner et al. supplementary material 1 — Turner et al. supplementary material [file S1368980025101298sup001.docx]

**Supplementary Material 1**

|  | **Policy** | **Practice** | **Provision** |
| --- | --- | --- | --- |
| **Qualitative (n=13)** | Private nurseries more isolated from healthy eating information, guided mainly by Office for Standards in Education (Ofsted) requirements^(1, 2)^.  Food policies implemented with varying degrees of flexibility^(3)^.  Government ‘30 hours free scheme’ resulted in ECEC relying on packed lunches or raising prices^(4)^.  Low number of ECEC had food policies^(2, 5)^.  ECEC willing to support breastfeeding but had no specific policy^(6)^.  Conflicting messages reported regarding policy for ‘treats’ and celebrations^(7)^.  Childminders viewed introduction of governmental monitoring as threatening and damaging to relationships between providers and Local Authorities (LA)^(8)^. | Staff and cooks had limited training in healthy eating for under-5s due to funding and inconsistent support^(1, 2, 4)^.  General healthy eating knowledge such as “five-a-day” relied upon^(3)^.  Use of colourful child-sized tableware to determine appropriate portion sizes^(9)^.  Parents reported minimal involvement and engagement with children’s food provision in ECEC^(7, 10)^.  Most common sources of information used were childminding magazines, internet and parents^(5)^.  Common practice to allow children to self-serve and give gentle encouragement to eat^(2, 9)^.  More emphasis on food hygiene than nutritional quality in childminders^(8)^.  Differences in vegetable consumption based on individual factors that affected acceptance^(11)^. | Quality of food provided largely governed by financial restraints, time capacity, and knowledge of what food to buy where^(1-3)^.  Settings that prepared own food had more autonomy over menus than ECEC using external provider, or reliant on school kitchens^(1, 5)^.  Over-reliance on fresh and dried fruit for snacks^(3)^.  Low proportion of children had milk at snack time^(12)^.  Introduction of Ofsted monitoring for childminders resulted in rise in childminders only serving packed lunches^(8)^.  Budgetary restraints were significant barrier in most deprived ECEC to provide healthier alternatives^(2)^. |
| **Survey & Questionnaire Analysis (n=8)** | Mixed attitudes from ECEC staff whether food guidelines should be statutory^(13)^.  ECEC that did not have food policy tended to exhibit less positive food practice and provision outcomes^(14)^. | Deprived nurseries more likely to align with recommended practices such as diluting juice^(15)^.  Higher levels of awareness than use of guidelines, higher awareness of Example Menus than Eat Better, Start Better (EBSB), but higher use of EBSB^(13)^.  21% practitioners agreed they use sweets and dessert as a reward for good behaviour, or to eat main meal^(16)^.  Majority of private nurseries prepared food on site, more LA funded used external food providers^(17)^.  ECEC reported menus were assessed for nutritional content, many said they received no specific healthy eating advice^(17)^.  Insufficient time, no kitchen facilities, and lack of staff were barriers to preparing and cooking food on site^(14)^.  Providers found it easiest to cater for nut intolerances and hardest to cater for Halal, Kosher and non-Beef Sikh meals^(14)^. | Meals provided in ECEC contained lower sugar and energy density than meals from parents, wider family and other childcare^(18)^.  ECEC in most deprived areas served more healthy foods, difference was driven by meeting guideline to serve wholegrains and legumes, pulses and lentils^(15)^.  ECEC furthest away from nearest supermarket more likely to serve fruit and veg less than 2-3 times per week^(19)^.  Average cost per child per main meal was £1.00-£2.00^(14, 17)^.  On average lunches were deficient in energy, carbohydrate, iron, zinc, and had excess salt^(17)^.  Settings with higher nutrition index scores had larger numbers of children, were in more deprived areas, and had greater proportion of staff from ethnic minority backgrounds^(14)^. |
| **Nutrition & Menu Analysis (n=3)** | Not reported. | Not reported. | Mean energy, fat, and carbohydrate content were above the nutrient framework. Mean free sugars was more than double recommended due to frequent provision of cakes and biscuits. Mean fibre and micronutrient content of lunches met nutrient framework^(20)^.  Most school-based nurseries provided same lunch food and portions to early years as Key Stage 1 pupils, lunches were more compliant with school standards than early years guidance^(20, 21)^. |
| **Experimental (n=3)** | Trial elements related to food policy did not improve^(22)^. | Children in nutrition education group had higher odds of consuming unfamiliar vegetable. Children in control group were least likely^(23)^.  Cost and time were identified as main barriers to implementing repeated taste exposures^(23)^. | Not reported. |

**Supplementary Table 1.** Summary of findings from included studies grouped by study design and Food Policy, Practice, and Provision concepts. Abbreviations: ECEC = Early Childhood Education and Care settings

| **Study** | **Individual** | **Interpersonal** | **Organisational** | **Environmental** | **Governmental** |
| --- | --- | --- | --- | --- | --- |
| Bristow et al., 2013 | N | Y | Y | Y | Y |
| Er et al., 2018 | Y | Y | Y | Y | N |
| Goldsborough, 2016 | Y | Y | Y | Y | N |
| Moore et al., 2005 | N | Y | Y | Y | Y |
| Alderton and Campbell-Barr, 2005 | N | Y | Y | Y | N |
| Marr et al., 2022 | N | Y | Y | Y | N |
| Neelon et al., 2014 | Y | N | Y | Y | N |
| Quirke-McFarlane et al., 2024 | Y | Y | Y | N | N |
| Warren et al., 2024 | N | N | Y | Y | Y |
| Warren et al., 2022 | N | Y | Y | N | Y |
| Williams et al., 2022 | Y | Y | Y | N | N |
| Albon, 2009 | Y | N | Y | N | N |
| Buttivant and Knai, 2012 | N | Y | Y | N | N |
| Dombrowski et al., 2020 | N | Y | Y | N | N |
| Langford et al., 2019 | N | N | Y | Y | N |
| Lloyd-Williams et al., 2011 | N | Y | Y | N | N |
| McLeod et al., 2023 | Y | N | Y | N | N |
| McSweeney et al., 2016 | N | Y | Y | N | N |
| Nekitsing et al., 2019 | Y | Y | N | N | N |
| Wall and Pearce, 2023 | N | N | Y | N | Y |
| Burgoine et al., 2017 | N | N | N | Y | N |
| Elford and Brown, 2014 | N | N | Y | N | N |
| Parker et al., 2011 | N | N | Y | N | N |
| Pearce and Wall, 2023 | N | N | Y | N | N |
| **Total** | **8** | **14** | **22** | **10** | **5** |

**Supplementary Table 2.** Allocations of SEM levels for each study.

*References*

1. Bristow K, Povall S, Capewell S *et al.* (2013) Exploring health inequalities through the lens of an ethnographic study of healthy eating provision in the early years sector. *Maternal & Child Nutrition* 9, 260-273.

2. Lloyd-Williams F, Bristow K, Capewell S *et al.* (2011) Young children's food in Liverpool day-care settings: a qualitative study of pre-school nutrition policy and practice. *Public Health Nutrition* 14, 1858-1866.

3. Goldsborough N, Homer C, Atchinson R *et al.* (2016) Healthy eating in the early years: A qualitative exploration of food provision in the childminder setting. *British Food Journal* 118, 992-1002.

4. Warren E, Williams L Knai C (2022) The "Cinderella sector": The challenges of promoting food and nutrition for young children in early years' settings in England. *Ecology of Food & Nutrition* 61, 576-594.

5. Buttivant H & Knai C (2012) Improving food provision in child care in England: a stakeholder analysis. *Public Health Nutrition* 15, 554-560.

6. Dombrowski L, Henderson S, Leslie J *et al.* (2020) The role of early years care providers in supporting continued breastfeeding and breast milk feeding. *Early Years: An International Journal of Research and Development* 40, 205-220.

7. McSweeney LA, Rapley T, Summerbell CD *et al.* (2016) Perceptions of nursery staff and parent views of healthy eating promotion in preschool settings: an exploratory qualitative study. *BMC Public Health* 16, 841.

8. Moore H, Nelson P, Marshall J *et al.* (2005) Laying foundations for health: food provision for under 5s in day care. *Appetite* 44, 207-213.

9. Quirke-McFarlane S, Carstairs SA Cecil JE (2024) 'You just eyeball it': Parent and nursery staff perceptions and influences on child portion size: A reflexive thematic analysis. *Nutrition and Health*, 2601060241245255.

10. Williams L, Warren E Knai C (2022) How involved are parents in their child's early years setting's food decisions and practices? *SSM Qualitative Research in Health* 2, 100142.

11. McLeod CJ, Haycraft E Daley AJ (2023) Offering vegetables to children at breakfast time in nursery and kindergarten settings: The Veggie Brek feasibility and acceptability cluster randomised controlled trial. *The International Journal of Behavioral Nutrition and Physical Activity Vol 20,(1), 2023, ArtID 38* 20.

12. Albon D (2009) Challenges to improving the uptake of milk in a nursery class: A case study. *Health Education* 109, 140-154.

13. Warren E, Boadu P, Exley J *et al.* (2024) Knowledge and use of voluntary food and drink guidelines in English nurseries? Results from a nationally representative cross-sectional study. *Food Policy* 122.

14. Alderton T & Campbell-Barr V (2005) Quality early education--quality food and nutrition practices? Some initial results from a pilot research project into food and nutrition practices in early years settings in Kent, UK. *International Journal of Early Years Education* 13, 197-213.

15. Neelon SE, Burgoine T, Hesketh KR *et al.* (2015) Nutrition practices of nurseries in England. Comparison with national guidelines. *Appetite* 85, 22-29.

16. Elford L & Brown A (2014) Exploring child-feeding style in childcare settings: How might nursery practitioners affect child eating style and weight? *Eating Behaviors* 15, 314-317.

17. Parker M, Lloyd-Williams F, Weston G *et al.* (2011) Nursery nutrition in Liverpool: an exploration of practice and nutritional analysis of food provided. *Public Health Nutrition* 14, 1867-1875.

18. Marr C, Breeze P Caton SJ (2022) Examination of dietary intake of UK preschool children by varying carers: evidence from the 2008-2016 UK National Diet and Nutrition Survey. *British Journal of Nutrition* 128, 2063-2074.

19. Burgoine T, Gallis JA, T LP *et al.* (2017) Association between distance to nearest supermarket and provision of fruits and vegetables in English nurseries. *Health & Place* 46, 229-233.

20. Wall CJ & Pearce J (2023) Energy and nutrient content of school lunches provided for children attending school-based nurseries: a cross-sectional study. *Public Health Nutrition* 26, 2641-2651.

21. Pearce J & Wall CJ (2023) School lunch portion sizes provided for children attending early years settings within primary schools: A cross-sectional study. *Journal of Human Nutrition & Dietetics* 36, 1887-1900.

22. Langford R, Jago R, White J *et al.* (2019) A physical activity, nutrition and oral health intervention in nursery settings: process evaluation of the NAP SACC UK feasibility cluster RCT. *BMC public health* 19, 865-865.

23. Nekitsing C, Blundell-Birtill P, Cockroft JE *et al.* (2019) Taste Exposure Increases Intake and Nutrition Education Increases Willingness to Try an Unfamiliar Vegetable in Preschool Children: A Cluster Randomized Trial. *Journal of the Academy of Nutrition and Dietetics* 119, 2004-2013.
