# Supplementary material for: Food policy, practice and provision in UK early childhood education and care: a scoping review
Source: Public Health Nutr. 2025 Oct 13;28(1):e176. doi: 10.1017/S1368980025101298 (PMC12722074; doi:10.1017/S1368980025101298)
Supplement: Turner et al. supplementary material 2 — Turner et al. supplementary material [file S1368980025101298sup002.docx]

**Supplementary Material 2**

**Ovid Medline**

|  | **Concept 1: Early years settings** |  |
| --- | --- | --- |
| 1 | Schools, Nursery/ | 1,512 |
| 2 | ("pre?school?" not "pre?schooler or pre?school child*").mp. | 1,005,469 |
| 3 | Child Day Care Centers/ | 5,354 |
| 4 | Children* centre.mp. | 87 |
| 5 | Child Care/ | 6,231 |
| 6 | (childcare* or child care*).mp. | 17,237 |
| 7 | (daycare* or day care*).mp. | 17,225 |
| 8 | (nursery or nurseries).mp. | 15,598 |
| 9 | Child$minder*.mp. | 42 |
| 10 | early year? setting*.mp. [mp=title, book title, abstract, original title, name of substance word, subject heading word, floating sub-heading word, keyword heading word, organism supplementary concept word, protocol supplementary concept word, rare disease supplementary concept word, unique identifier, synonyms, population supplementary concept word, anatomy supplementary concept word] | 31 |
| 11 | 1 or 2 or 3 or 4 or 5 or 6 or 7 or 8 or 9 or 10 | 1,038,766 |
|  | **Concept 2: Early Years children** |  |
| 12 | (child* or boys or girls or kids or paediatric* or pediatric* or young).mp. | 4,117,161 |
| 13 | early child*.mp. | 37,680 |
| 14 | ("0 years old" or 0-1 years old or 0-2 years old or 0-3 years old or 0-4 years old or 0-5 years old or 1 years old or 1-2 years old or 1-3 years old or 1-4 years old or 1-5 years old or 2 years old or 2-3 years old or 2-4 years old or 2-5 years old or 3 years old or 3-4 years old or 3-5 years old or 4 years old or 4-5 years old or 5 years old).mp. | 16,438 |
| 15 | pre?school*.mp. | 1,006,854 |
| 16 | exp Child, Preschool/ | 998,391 |
| 17 | 12 or 13 or 14 or 15 or 16 | 4,120,288 |
| 18 | 11 and 17 | 1,022,243 |
| 19 | 11 or 18 | 1,038,766 |
|  | **Concept 3: Food nutrition, feeding practices, & food events** |  |
| 20 | diet/ or diet composition/ or unhealthy diet/ or healthy diet/ | 197,425 |
| 21 | Energy intake/ or energy intake.mp. | 58,987 |
| 22 | Child nutrition.mp. or child nutrition/ | 7,348 |
| 23 | nutritional parameters/ or nutritional health/ or nutritional requirement/ | 20,134 |
| 24 | feeding behavio$r.mp. or feeding behavior/ | 98,703 |
| 25 | Dietary intake.mp. or dietary intake/ | 90,169 |
| 26 | (nutritional intake or nutrient intake or food intake).mp. | 65,027 |
| 27 | Dietary pattern.mp. or dietary pattern/ | 6,572 |
| 28 | Nutritional status.mp. or nutritional status/ | 80,810 |
| 29 | ((diet* adj2 (quality* or intake* or behavior* or behaviour* or pattern*)) or (nutrition* adj2 (behaviour* or behavior* or status* or knowledg* or skill*)) or (food* adj2 (pattern* or habit* or quality* or frequenc* or preferenc* or knowledg* or skill* or belief* or attitude* or provision*)) or ((eating* or feeding*) adj2 (habit* or practic* or pattern*))).mp. | 224,374 |
| 30 | "Diet, Food, and Nutrition"/ | 3 |
| 31 | 20 or 21 or 22 or 23 or 24 or 25 or 26 or 27 or 28 or 29 or 30 | 537,116 |
| 32 | Food event.mp. | 8 |
| 33 | (meal* or lunch* or snack).mp. | 97,189 |
| 34 | Eating.mp. or Eating/ | 160,433 |
| 35 | 32 or 33 or 34 | 239,290 |
|  | **Concept 4: food policy & guidelines** |  |
| 36 | "food policy".mp. or Nutrition Policy/ | 11,470 |
| 37 | Guideline/ or practice guideline/ | 38,294 |
| 38 | (Nutritional guidance or nutritional guideline* or food guidance or food guideline* or diet guidance or diet guideline*).mp. | 1,235 |
| 39 | 36 or 37 or 38 | 50,705 |
| 40 | 31 or 35 or 39 | 693,533 |
| 41 | 19 and 40 | 38,668 |
|  | **Limit to UK Countries** |  |
| 42 | exp United Kingdom/ | 393,772 |
| 43 | (UK or United Kingdom or Britain or Wales or Scotland or Northern Ireland or England or English or Scottish or Welsh or Irish).mp. [mp=title, book title, abstract, original title, name of substance word, subject heading word, floating sub-heading word, keyword heading word, organism supplementary concept word, protocol supplementary concept word, rare disease supplementary concept word, unique identifier, synonyms, population supplementary concept word, anatomy supplementary concept word] | 2,250,999 |
| 44 | (national health service* or nhs*).ti,ab,in. | 288,594 |
| 45 | (english not ((published or publication* or translat* or written or language* or speak* or literature or citation*) adj5 english)).ti,ab. | 126,261 |
| 46 | (gb or "g.b." or britain* or (british* not "british columbia") or uk or "u.k." or united kingdom* or (england* not "new england") or northern ireland* or northern irish* or scotland* or scottish* or ((wales or "south wales") not "new south wales") or welsh*).ti,ab,jw,in. | 2,538,950 |
| 47 | (bath or "bath's" or ((birmingham not alabama*) or ("birmingham's" not alabama*) or bradford or "bradford's" or brighton or "brighton's" or bristol or "bristol's" or carlisle* or "carlisle's" or (cambridge not (massachusetts* or boston* or harvard*)) or ("cambridge's" not (massachusetts* or boston* or harvard*)) or (canterbury not zealand*) or ("canterbury's" not zealand*) or chelmsford or "chelmsford's" or chester or "chester's" or chichester or "chichester's" or coventry or "coventry's" or derby or "derby's" or (durham not (carolina* or nc)) or ("durham's" not (carolina* or nc)) or ely or "ely's" or exeter or "exeter's" or gloucester or "gloucester's" or hereford or "hereford's" or hull or "hull's" or lancaster or "lancaster's" or leeds* or leicester or "leicester's" or (lincoln not nebraska*) or ("lincoln's" not nebraska*) or (liverpool not (new south wales* or nsw)) or ("liverpool's" not (new south wales* or nsw)) or ((london not (ontario* or ont or toronto*)) or ("london's" not (ontario* or ont or toronto*)) or manchester or "manchester's" or (newcastle not (new south wales* or nsw)) or ("newcastle's" not (new south wales* or nsw)) or norwich or "norwich's" or nottingham or "nottingham's" or oxford or "oxford's" or peterborough or "peterborough's" or plymouth or "plymouth's" or portsmouth or "portsmouth's" or preston or "preston's" or ripon or "ripon's" or salford or "salford's" or salisbury or "salisbury's" or sheffield or "sheffield's" or southampton or "southampton's" or st albans or stoke or "stoke's" or sunderland or "sunderland's" or truro or "truro's" or wakefield or "wakefield's" or wells or westminster or "westminster's" or winchester or "winchester's" or wolverhampton or "wolverhampton's" or (worcester not (massachusetts* or boston* or harvard*)) or ("worcester's" not (massachusetts* or boston* or harvard*)) or (york not ("new york*" or ny or ontario* or ont or toronto*)) or ("york's" not ("new york*" or ny or ontario* or ont or toronto*))))).ti,ab,in. | 1,826,891 |
| 48 | (bangor or "bangor's" or cardiff or "cardiff's" or newport or "newport's" or st asaph or "st asaph's" or st davids or swansea or "swansea's").ti,ab,in. | 73,868 |
| 49 | (aberdeen or "aberdeen's" or dundee or "dundee's" or edinburgh or "edinburgh's" or glasgow or "glasgow's" or inverness or (perth not australia*) or ("perth's" not australia*) or stirling or "stirling's").ti,ab,in. | 268,766 |
| 50 | (armagh or "armagh's" or belfast or "belfast's" or lisburn or "lisburn's" or londonderry or "londonderry's" or derry or "derry's" or newry or "newry's").ti,ab,in. | 35,637 |
| 51 | 42 or 43 or 44 or 45 or 46 or 47 or 48 or 49 or 50 | 4,844,783 |
| 52 | (exp africa/ or exp americas/ or exp antarctic regions/ or exp arctic regions/ or exp asia/ or exp australia/ or exp oceania/) not (exp United Kingdom/ or europe/) | 3,405,883 |
| 53 | 51 not 52 | 4,521,682 |
|  | **Limit Study Design** |  |
| 54 | "systematic review".pt. or "Systematic Reviews as Topic"/ or "Cochrane Database of Systematic Reviews".jn. or (evidence report technology assessment or evidence report technology assessment summary).jn. or (((comprehensive* or integrative or mapping or rapid or realist or scoping or systematic or systematical or systematically or systematicaly or systematicly or umbrella) adj3 (bibliographical or bibliographically or bibliographics or literature or review or reviews)) or (state adj3 art adj1 review) or (research adj2 synthesis) or ((data or information) adj3 synthesis)).ti,ab,kf. or ((data adj2 (extract or extracting or extractings or extraction or extraction)).ti,ab,kf. and ("review".ti. or "review".pt.)) or (((electronic or searched) adj2 database*) and (eligibility or excluded or exclusion or included or inclusion)).ti,ab,kf. or (overview adj4 reviews).ti,ab,kf. or ((review adj3 (rationale or evidence)).ti,ab. and "review".pt.) or (PRISMA or (preferred adj1 reporting)).ab. or (cinahl or (cochrane adj3 (trial or trials)) or embase or medline or psyclit or (psycinfo not (psycinfo adj1 database)) or pubmed or scopus or (sociological adj1 abstracts) or (web adj2 science)).ab. | 640,603 |
| 55 | protocol.ti. and (Systematic Review.pt. or Systematic Reviews as Topic/ or (((comprehensive or critical or exploratory or Integrated or integrative or mapping or methodology or narrative or prognostic or psychometric or qualitative or quantitative or rapid or (review adj1 reviews) or realist or scoping or (state adj3 art) or systematic or umbrella) adj2 (review or reviews or search or searches)) or (evidence adj1 (assessment or mapping)) or (framework adj1 synthesis) or ((meta or (mixed adj1 methods)) adj1 (review or synthesis))).ti.) | 11,749 |
| 56 | 54 or 55 | 640,629 |
| 57 | 41 and 53 | 4,746 |
| 58 | 57 not 56 | 4,371 |
| 59 | exp animal experiment/ or exp animal model/ or exp transgenic animal/ or animal/ or exp amphibia/ or mammal/ or exp reptile/ or exp marsupial/ or primate/ or pig*.mp. | 7,602,618 |
| 60 | Maternal Behavior/ or Maternal Age/ or maternal.mp. or Maternal Health/ | 384,320 |
| 61 | 58 not (59 or 60) | 3,685 |
| 62 | limit 61 to (english language and yr="1990 -Current") | 2,374 |
|  | Search 17/04/2024 |  |

**Ovid Embase**

| **#** | **Query** | **Results from 17 Apr 2024** |
| --- | --- | --- |
| 1 | nursery school/ or nursery/ | 5,348 |
| 2 | ("pre?school?" not "pre?schooler or pre?school child*").mp. | 648,846 |
| 3 | child day care/ | 141 |
| 4 | Children* centre.mp. | 151 |
| 5 | Child Care/ | 40,734 |
| 6 | (childcare* or child care*).mp. | 48,610 |
| 7 | (daycare* or day care*).mp. | 19,615 |
| 8 | (nursery or nurseries).mp. | 15,420 |
| 9 | Child$minder*.mp. | 50 |
| 10 | early year? setting*.mp. | 50 |
| 11 | 1 or 2 or 3 or 4 or 5 or 6 or 7 or 8 or 9 or 10 | 716,280 |
| 12 | (child* or boys or girls or kids or paediatric* or pediatric* or young).mp. | 4,421,130 |
| 13 | early child*.mp. | 49,053 |
| 14 | ("0 years old" or 0-1 years old or 0-2 years old or 0-3 years old or 0-4 years old or 0-5 years old or 1 years old or 1-2 years old or 1-3 years old or 1-4 years old or 1-5 years old or 2 years old or 2-3 years old or 2-4 years old or 2-5 years old or 3 years old or 3-4 years old or 3-5 years old or 4 years old or 4-5 years old or 5 years old).mp. | 26,897 |
| 15 | pre?school*.mp. | 650,387 |
| 16 | exp preschool child/ | 636,311 |
| 17 | 12 or 13 or 14 or 15 or 16 | 4,426,532 |
| 18 | 11 and 17 | 698,559 |
| 19 | 11 or 18 | 716,280 |
| 20 | diet composition/ or diet/ | 261,396 |
| 21 | energy intake.mp. or caloric intake/ | 87,148 |
| 22 | child nutrition.mp. or child nutrition/ | 20,562 |
| 23 | nutritional parameters/ or nutritional health/ or nutritional requirement/ | 31,013 |
| 24 | feeding behavio$r.mp. or feeding behavior/ | 100,154 |
| 25 | dietary intake.mp. or dietary intake/ | 118,872 |
| 26 | (nutritional intake or nutrient intake or food intake).mp. | 196,777 |
| 27 | dietary pattern.mp. or dietary pattern/ | 14,372 |
| 28 | nutritional status.mp. or nutritional status/ | 101,710 |
| 29 | ((diet* adj2 (quality* or intake* or behavior* or behaviour* or pattern*)) or (nutrition* adj2 (behaviour* or behavior* or status* or knowledg* or skill*)) or (food* adj2 (pattern* or habit* or quality* or frequenc* or preferenc* or knowledg* or skill* or belief* or attitude* or provision*)) or ((eating* or feeding*) adj2 (habit* or practic* or pattern*))).mp. | 357,725 |
| 30 | food/ | 80,913 |
| 31 | 20 or 21 or 22 or 23 or 24 or 25 or 26 or 27 or 28 or 29 or 30 | 865,694 |
| 32 | food event.mp. | 11 |
| 33 | (meal* or lunch* or snack).mp. | 138,668 |
| 34 | eating.mp. or eating/ | 168,147 |
| 35 | 32 or 33 or 34 | 286,820 |
| 36 | nutrition policy/ or food policy.mp. | 3,670 |
| 37 | (Nutritional guidance or nutritional guideline* or food guidance or food guideline* or diet guidance or diet guideline*).mp. | 1,853 |
| 38 | 36 or 37 | 5,473 |
| 39 | 31 or 35 or 38 | 1,023,666 |
| 40 | 19 and 39 | 35,930 |
| 41 | exp United Kingdom/ | 474,038 |
| 42 | (UK or United Kingdom or Britain or Wales or Scotland or Northern Ireland or England or English or Scottish or Welsh or Irish).mp. [mp=title, abstract, heading word, drug trade name, original title, device manufacturer, drug manufacturer, device trade name, keyword heading word, floating subheading word, candidate term word] | 1,082,674 |
| 43 | (national health service* or nhs*).ti,ab,in. | 425,979 |
| 44 | (english not ((published or publication* or translat* or written or language* or speak* or literature or citation*) adj5 english)).ti,ab. | 64,017 |
| 45 | (gb or "g.b." or britain* or (british* not "british columbia") or uk or "u.k." or united kingdom* or (england* not "new england") or northern ireland* or northern irish* or scotland* or scottish* or ((wales or "south wales") not "new south wales") or welsh*).ti,ab,jw,in. | 3,748,626 |
| 46 | (bath or "bath's" or ((birmingham not alabama*) or ("birmingham's" not alabama*) or bradford or "bradford's" or brighton or "brighton's" or bristol or "bristol's" or carlisle* or "carlisle's" or (cambridge not (massachusetts* or boston* or harvard*)) or ("cambridge's" not (massachusetts* or boston* or harvard*)) or (canterbury not zealand*) or ("canterbury's" not zealand*) or chelmsford or "chelmsford's" or chester or "chester's" or chichester or "chichester's" or coventry or "coventry's" or derby or "derby's" or (durham not (carolina* or nc)) or ("durham's" not (carolina* or nc)) or ely or "ely's" or exeter or "exeter's" or gloucester or "gloucester's" or hereford or "hereford's" or hull or "hull's" or lancaster or "lancaster's" or leeds* or leicester or "leicester's" or (lincoln not nebraska*) or ("lincoln's" not nebraska*) or (liverpool not (new south wales* or nsw)) or ("liverpool's" not (new south wales* or nsw)) or ((london not (ontario* or ont or toronto*)) or ("london's" not (ontario* or ont or toronto*)) or manchester or "manchester's" or (newcastle not (new south wales* or nsw)) or ("newcastle's" not (new south wales* or nsw)) or norwich or "norwich's" or nottingham or "nottingham's" or oxford or "oxford's" or peterborough or "peterborough's" or plymouth or "plymouth's" or portsmouth or "portsmouth's" or preston or "preston's" or ripon or "ripon's" or salford or "salford's" or salisbury or "salisbury's" or sheffield or "sheffield's" or southampton or "southampton's" or st albans or stoke or "stoke's" or sunderland or "sunderland's" or truro or "truro's" or wakefield or "wakefield's" or wells or westminster or "westminster's" or winchester or "winchester's" or wolverhampton or "wolverhampton's" or (worcester not (massachusetts* or boston* or harvard*)) or ("worcester's" not (massachusetts* or boston* or harvard*)) or (york not ("new york*" or ny or ontario* or ont or toronto*)) or ("york's" not ("new york*" or ny or ontario* or ont or toronto*))))).ti,ab,in. | 2,978,217 |
| 47 | (bangor or "bangor's" or cardiff or "cardiff's" or newport or "newport's" or st asaph or "st asaph's" or st davids or swansea or "swansea's").ti,ab,in. | 122,893 |
| 48 | (aberdeen or "aberdeen's" or dundee or "dundee's" or edinburgh or "edinburgh's" or glasgow or "glasgow's" or inverness or (perth not australia*) or ("perth's" not australia*) or stirling or "stirling's").ti,ab,in. | 410,342 |
| 49 | (armagh or "armagh's" or belfast or "belfast's" or lisburn or "lisburn's" or londonderry or "londonderry's" or derry or "derry's" or newry or "newry's").ti,ab,in. | 57,439 |
| 50 | 41 or 42 or 43 or 44 or 45 or 46 or 47 or 48 or 49 | 4,845,273 |
| 51 | (exp africa/ or exp americas/ or exp antarctic regions/ or exp arctic regions/ or exp asia/ or exp australia/ or exp oceania/) not (exp United Kingdom/ or europe/) | 3,743,871 |
| 52 | 50 not 51 | 4,537,336 |
| 53 | "systematic review".pt. or "Systematic Reviews as Topic"/ or "Cochrane Database of Systematic Reviews".jn. or (evidence report technology assessment or evidence report technology assessment summary).jn. or (((comprehensive* or integrative or mapping or rapid or realist or scoping or systematic or systematical or systematically or systematicaly or systematicly or umbrella) adj3 (bibliographical or bibliographically or bibliographics or literature or review or reviews)) or (state adj3 art adj1 review) or (research adj2 synthesis) or ((data or information) adj3 synthesis)).ti,ab,kf. or ((data adj2 (extract or extracting or extractings or extraction or extraction)).ti,ab,kf. and ("review".ti. or "review".pt.)) or (((electronic or searched) adj2 database*) and (eligibility or excluded or exclusion or included or inclusion)).ti,ab,kf. or (overview adj4 reviews).ti,ab,kf. or ((review adj3 (rationale or evidence)).ti,ab. and "review".pt.) or (PRISMA or (preferred adj1 reporting)).ab. or (cinahl or (cochrane adj3 (trial or trials)) or embase or medline or psyclit or (psycinfo not (psycinfo adj1 database)) or pubmed or scopus or (sociological adj1 abstracts) or (web adj2 science)).ab. | 789,470 |
| 54 | protocol.ti. and (Systematic Review.pt. or Systematic Reviews as Topic/ or (((comprehensive or critical or exploratory or Integrated or integrative or mapping or methodology or narrative or prognostic or psychometric or qualitative or quantitative or rapid or (review adj1 reviews) or realist or scoping or (state adj3 art) or systematic or umbrella) adj2 (review or reviews or search or searches)) or (evidence adj1 (assessment or mapping)) or (framework adj1 synthesis) or ((meta or (mixed adj1 methods)) adj1 (review or synthesis))).ti.) | 11,379 |
| 55 | 53 or 54 | 789,502 |
| 56 | 40 and 52 | 3,511 |
| 57 | 56 not 55 | 3,250 |
| 58 | exp animal experiment/ or exp animal model/ or exp transgenic animal/ or animal/ or exp amphibia/ or mammal/ or exp reptile/ or exp marsupial/ or primate/ or pig*.mp. | 5,547,915 |
| 59 | Maternal Behavior/ or Maternal Age/ or maternal.mp. or Maternal Health/ | 499,709 |
| 60 | 57 not (58 or 59) | 2,770 |
| 61 | limit 60 to (english language and yr="1990 -Current") | 2,579 |

**Ovid PsychINFO**

|  | **Concept 1: Early years settings** |  |
| --- | --- | --- |
| 1 | Nursery Schools/ | 507 |
| 2 | pre?school*.mp. | 118,466 |
| 3 | Day Care Centers/ or Child Day Care/ | 3,482 |
| 4 | Children* centre.mp. | 65 |
| 5 | Child Care/ | 8,902 |
| 6 | (childcare* or child care*).mp. | 21,355 |
| 7 | (daycare* or day care*).mp. | 9,212 |
| 8 | (nursery or nurseries).mp. | 5,540 |
| 9 | Child$minder*.mp. | 37 |
| 10 | early year? setting*.mp. | 178 |
| 11 | 1 or 2 or 3 or 4 or 5 or 6 or 7 or 8 or 9 or 10 | 145,053 |
|  | **Concept 2: Early Years children** |  |
| 12 | (child* or boys or girls or kids or paediatric* or pediatric* or young).mp. | 1,236,718 |
| 13 | early child*.mp. | 45,593 |
| 14 | ("0 years old" or 0-1 years old or 0-2 years old or 0-3 years old or 0-4 years old or 0-5 years old or 1 years old or 1-2 years old or 1-3 years old or 1-4 years old or 1-5 years old or 2 years old or 2-3 years old or 2-4 years old or 2-5 years old or 3 years old or 3-4 years old or 3-5 years old or 4 years old or 4-5 years old or 5 years old).mp. | 2,541 |
| 15 | kinder*.mp. | 30,889 |
| 16 | exp Preschool Students/ | 14,131 |
| 17 | 12 or 13 or 14 or 15 or 16 | 1,242,871 |
| 18 | 11 and 17 | 138,864 |
| 19 | 11 or 18 | 145,053 |
|  | **Concept 3: Food nutrition, feeding practices, & food events** |  |
| 20 | exp Diets/ | 21,030 |
| 21 | Food Intake/ or energy intake.mp. | 19,321 |
| 22 | Nutrition/ | 13,589 |
| 23 | (nutritional requirement or nutritional health).mp. [mp=title, abstract, heading word, table of contents, key concepts, original title, tests & measures, mesh word] | 181 |
| 24 | Eating Behavior/ or eating behaviour.mp. | 18,293 |
| 25 | dietary intake.mp. | 2,883 |
| 26 | (nutritional intake or nutrient intake or food intake).mp. | 22,080 |
| 27 | dietary pattern.mp. | 472 |
| 28 | nutritional status.mp. | 3,545 |
| 29 | ((diet* adj2 (quality* or intake* or behavior* or behaviour* or pattern*)) or (nutrition* adj2 (behaviour* or behavior* or status* or knowledg* or skill*)) or (food* adj2 (pattern* or habit* or quality* or frequenc* or preferenc* or knowledg* or skill* or belief* or attitude* or provision*)) or ((eating* or feeding*) adj2 (habit* or practic* or pattern*))).mp. | 37,360 |
| 30 | Food/ | 15,474 |
| 31 | 20 or 21 or 22 or 23 or 24 or 25 or 26 or 27 or 28 or 29 or 30 | 89,002 |
| 32 | Food event.mp. | 16 |
| 33 | (meal* or lunch* or snack).mp. | 17,324 |
| 34 | eating.mp. or exp Eating Behavior/ | 84,026 |
| 35 | 32 or 33 or 34 | 94,321 |
|  | **Concept 4: food policy & guidelines** |  |
| 36 | food policy.mp. | 197 |
| 37 | practice guideline.mp. | 935 |
| 38 | (Nutritional guidance or nutritional guideline* or food guidance or food guideline* or diet guidance or diet guideline*).mp. | 155 |
| 39 | 36 or 37 or 38 | 1,285 |
| 40 | 31 or 35 or 39 | 143,453 |
| 41 | 19 and 40 | 5,453 |
|  | **Limit to UK Countries** |  |
| 42 | United Kingdom.mp. | 16,604 |
| 43 | (UK or United Kingdom or Britain or Wales or Scotland or Northern Ireland or England or English or Scottish or Welsh or Irish).mp. | 273,318 |
| 44 | (national health service* or nhs*).ti,ab,in. | 30,745 |
| 45 | (english not ((published or publication* or translat* or written or language* or speak* or literature or citation*) adj5 english)).ti,ab. | 102,877 |
| 46 | (gb or "g.b." or britain* or (british* not "british columbia") or uk or "u.k." or united kingdom* or (england* not "new england") or northern ireland* or northern irish* or scotland* or scottish* or ((wales or "south wales") not "new south wales") or welsh*).ti,ab,jw,in. | 539,161 |
| 47 | (bath or "bath's" or ((birmingham not alabama*) or ("birmingham's" not alabama*) or bradford or "bradford's" or brighton or "brighton's" or bristol or "bristol's" or carlisle* or "carlisle's" or (cambridge not (massachusetts* or boston* or harvard*)) or ("cambridge's" not (massachusetts* or boston* or harvard*)) or (canterbury not zealand*) or ("canterbury's" not zealand*) or chelmsford or "chelmsford's" or chester or "chester's" or chichester or "chichester's" or coventry or "coventry's" or derby or "derby's" or (durham not (carolina* or nc)) or ("durham's" not (carolina* or nc)) or ely or "ely's" or exeter or "exeter's" or gloucester or "gloucester's" or hereford or "hereford's" or hull or "hull's" or lancaster or "lancaster's" or leeds* or leicester or "leicester's" or (lincoln not nebraska*) or ("lincoln's" not nebraska*) or (liverpool not (new south wales* or nsw)) or ("liverpool's" not (new south wales* or nsw)) or ((london not (ontario* or ont or toronto*)) or ("london's" not (ontario* or ont or toronto*)) or manchester or "manchester's" or (newcastle not (new south wales* or nsw)) or ("newcastle's" not (new south wales* or nsw)) or norwich or "norwich's" or nottingham or "nottingham's" or oxford or "oxford's" or peterborough or "peterborough's" or plymouth or "plymouth's" or portsmouth or "portsmouth's" or preston or "preston's" or ripon or "ripon's" or salford or "salford's" or salisbury or "salisbury's" or sheffield or "sheffield's" or southampton or "southampton's" or st albans or stoke or "stoke's" or sunderland or "sunderland's" or truro or "truro's" or wakefield or "wakefield's" or wells or westminster or "westminster's" or winchester or "winchester's" or wolverhampton or "wolverhampton's" or (worcester not (massachusetts* or boston* or harvard*)) or ("worcester's" not (massachusetts* or boston* or harvard*)) or (york not ("new york*" or ny or ontario* or ont or toronto*)) or ("york's" not ("new york*" or ny or ontario* or ont or toronto*))))).ti,ab,in. | 420,357 |
| 48 | (bangor or "bangor's" or cardiff or "cardiff's" or newport or "newport's" or st asaph or "st asaph's" or st davids or swansea or "swansea's").ti,ab,in. | 22,031 |
| 49 | (aberdeen or "aberdeen's" or dundee or "dundee's" or edinburgh or "edinburgh's" or glasgow or "glasgow's" or inverness or (perth not australia*) or ("perth's" not australia*) or stirling or "stirling's").ti,ab,in. | 51,972 |
| 50 | (armagh or "armagh's" or belfast or "belfast's" or lisburn or "lisburn's" or londonderry or "londonderry's" or derry or "derry's" or newry or "newry's").ti,ab,in. | 7,124 |
| 51 | 42 or 43 or 44 or 45 or 46 or 47 or 48 or 49 or 50 | 760,776 |
| 52 | ((africa or americas or antarctic regions or arctic regions or asia or australia or oceania) not (United Kingdom or europe)).mp. [mp=title, abstract, heading word, table of contents, key concepts, original title, tests & measures, mesh word] | 87,060 |
| 53 | 51 not 52 | 744,400 |
| 54 | 41 and 53 | 851 |
| 55 | limit 54 to (english language and yr="1990 -Current") | 740 |
|  | Search 09/04/2024 |  |

**CINAHL**

|  | **Concept 1: Early years settings** |  |
| --- | --- | --- |
| 1 | (MH "Child Care") OR (MH "Schools, Nursery") OR (MH "Child, Preschool") OR (MH "Child Day Care") OR "nursery or preschool or early years or kindergarten or childcare" | 237,070 |
| 2 | "childminder*" | 21 |
| 3 | "early year? setting*" | 1 |
|  | **Concept 2: Early Years children** |  |
| 4 | (MH "Child, Preschool") OR "young children or early childhood or preschool or kindergarten or early years" | 231,810 |
| 5 | S1 OR S2 OR S3 | 237,076 |
| 6 | S4 AND S5 | 231,810 |
|  | **Concept 3: Food nutrition, feeding practice, & nutrition policy** |  |
| 7 | (MH "Food Habits") OR (MH "Nutrition Policy") OR (MH "Nutritional Status: Food & Fluid Intake (Iowa NOC)") OR (MH "Nutritive Value") OR "diet and nutrition or eating habit or food" OR (MH "Eating") OR (MH "Diet") OR (MH "Eating Behavior") OR (MH "Nutrition") | 128,450 |
| 8 | (MH "Child Nutrition") OR "child nutrition" | 9,596 |
| 9 | (MH "Nutritional Status: Nutrient Intake (Iowa NOC)") OR (MH "Nutritional Status: Food & Fluid Intake (Iowa NOC)") OR (MH "Dietary Reference Intakes") OR "dietary intake or nutritional intake or dietary nutrition" | 5,251 |
| 10 | S7 OR S8 OR S9 | 138,071 |
| 11 | (MH "Meals") OR (MH "Breakfast") OR (MH "Lunch") OR (MH "Snacks") | 12,011 |
| 12 | S10 OR S11 | 144,578 |
| 13 | (S10 OR S11) AND (S5 AND S6 AND S12) | 10,088 |
| 55 | \| Limit to 1990 to 2024 \| \| --- \| \| Source types: Academic journals \| \| Language: English \| \| Geography: uk & ireland \| | 2,754 |
|  | Search 09/04/2024 |  |

**Scopus**

|  |  |  |
| --- | --- | --- |
| 1 | ( TITLE-ABS-KEY ( diet* OR "diet*composition" OR "energy intake" OR "child*nutrition" OR "healthy diet" OR "diet*intake" OR "food behavio?r" OR "food habit*" OR "feeding practice*" OR "diet pattern" OR "food event" OR "mealtime" OR eating OR lunch* OR "food policy" OR "nutrition policy" OR "nutrition*guidance" OR " food?guid*" ) ) AND ( ( TITLE-ABS-KEY ( nurser* OR pre?school OR "day care centre*" OR {early year? setting*}   OR child?minder* ) AND TITLE-ABS-KEY ( "early?year*" OR child* OR "early?child*" OR kid* ) ) OR ( TITLE-ABS-KEY ( nurser* OR pre?school OR "day care centre*" OR {early year? setting*} OR child?minder* ) ) ) |  |
| 2 | ( TITLE-ABS-KEY ( diet* OR "diet*composition" OR "energy intake" OR "child*nutrition" OR "healthy diet" OR "diet*intake" OR "food behavio?r" OR "food habit*" OR "feeding practice*" OR "diet pattern" OR "food event" OR "mealtime" OR eating OR lunch* OR "food policy" OR "nutrition policy" OR "nutrition*guidance" OR " food?guid*" ) ) AND ( ( TITLE-ABS-KEY ( nurser* OR pre?school OR "day care centre*" OR {early year? setting*} OR child?minder* ) AND TITLE-ABS-KEY ( "early?year*" OR child* OR "early?child*" OR kid* ) ) OR ( TITLE-ABS-KEY ( nurser* OR pre?school OR "day care centre*" OR {early year? setting*} OR child?minder* ) ) ) AND PUBYEAR > 1989 AND PUBYEAR < 2025 AND ( LIMIT-TO ( DOCTYPE , "ar" ) ) AND ( LIMIT-TO ( LANGUAGE , "english" ) ) | 2,142 |
|  | Search 09/04/2024 |  |
